# Focus on PNA Flexibility and RNA Binding using Molecular Dynamics and Metadynamics

**DOI:** 10.1038/srep42799

**Published:** 2017-02-17

**Authors:** Massimiliano Donato Verona, Vincenzo Verdolino, Ferruccio Palazzesi, Roberto Corradini

**Affiliations:** 1 Dipartimento di Chimica, University of Parma, Italy, 43124, Italy; 2 Department of Chemistry and Applied Biosciences, ETH Zurich, c/o Università della Svizzera Italiana Campus, 6900 Lugano, Switzerland; 3 Facoltà di Informatica, Instituto di Scienze Computazionali, Università della Svizzera Italiana, 6900 Lugano, Switzerland; 4 National Institute for Biostructures and Biosystems (INBB)-Viale delle Medaglie d’Oro, 305, 00136 Roma, Italy

## Abstract

Peptide Nucleic Acids (PNAs) can efficiently target DNA or RNA acting as chemical tools for gene regulation. Their backbone modification and functionalization is often used to increase the affinity for a particular sequence improving selectivity. The understanding of the trading forces that lead the single strand PNA to bind the DNA or RNA sequence is preparatory for any further rational design, but a clear and unique description of this process is still not complete. In this paper we report further insights into this subject, by a computational investigation aiming at the characterization of the conformations of a single strand PNA and how these can be correlated to its capability in binding DNA/RNA. Employing Metadynamics we were able to better define conformational pre-organizations of the single strand PNA and *γ*-modified PNA otherwise unrevealed through classical molecular dynamics. Our simulations driven on backbone modified PNAs lead to the conclusion that this *γ*-functionalization affects the single strand preorganization and targeting properties to the DNA/RNA, in agreement with circular dichroism (CD) spectra obtained for this class of compounds. MD simulations on PNA:RNA dissociation and association mechanisms allowed to reveal the critical role of central bases and preorganization in the binding process.

The design of chemical tools for gene regulation is one of the most promising approaches to the development of new drugs. Among the variety of molecules proposed for this scope, Peptide Nucleic Acids (PNAs) are one of the most established and efficient artificial systems for targeting DNA or RNA so far described; these compounds are nucleic acid analogues in which the deoxyribose phosphate backbone is substituted by a polyamidic chain of N-(2-aminoethyl)-glycine units (Fig. 1a)^1^,^2^. Due to their high affinity for DNA and RNA, and to the high sequence-selectivity of their interaction with complementary nucleic acids, PNAs are widely used as probes for the recognition of specific DNA sequences and, conjugated to surfaces or to reporter groups, for diagnostic application or imaging^2^,^3^,^4^,^5^. In drug development, PNAs have been used as anti-gene^6^,^7^,^8^, (i.e. blocking transcription of genomic DNA), as antisense agents^9^,^10^,^11^,^12^, (i.e. preventing translation of mRNA into proteins) and were found to be able to redirect aberrant splicing^13^.

An emerging challenging application is the use of PNA targeting micro-RNA (miR). Up- or down-regulation of miRs has been correlated with many different diseases, including cancer^14^, and thus the anti-miR targeting has become more and more important in drug development^15^,^16^,^17^ and in diagnostics^18^,^19^. Anti-miR PNA have been used for modulating differentiation^20^, arrest cellular proliferation^21^,^22^, and inducing apoptosis^23^. Detection of miRs in living cells has also been achieved by means of PNA probes^24^.

Many PNAs with modified backbones^25^,^26^ or modified nucleobases^27^ have been reported, in the continuous quest for further improvement of their affinity and selectivity for DNA and RNA. It is known that modifications on the backbone are useful for modulating the stability and sequence-selectivity in the formation of PNA:DNA or PNA:RNA duplexes^25^,^28^,^29^, but also for the introduction of functional moieties, such as positively charged groups, fluorophores, or linkers for surface tethering^30^,^31^. Interestingly, backbone modifications were shown to differently affect helicity and DNA binding^28^. In particular, modifications in the *γ*-position (Fig. 1b) were shown to induce a preferred chiral single strand PNA conformation by circular dichroism (CD) experiments, unlike the unmodified or *α*-modified PNAs^28^,^29^. The CD signatures of these modified PNAs have been correlated to the presence of both nucleobase and backbone carbonyl groups presenting a preferred helical orientation as a result of a “preorganization” induced by substitution in the *γ*-position^29^. This feature increases PNA affinity and selectivity toward targets^32^, and enables the formation of more stable duplexes^29^, thus favoring DNA double strand invasion^33^. The positioning of modified monomers, as well as that of mismatches, strongly affects the binding properties and selectivity of PNA, and a complete picture of the effect of modification and of positioning of the bases is still far from being established. In a general project aimed to use PNA in the modulation and detection of tumor-related micro-RNAs, we intend to rationally design new modified PNA, in particular those able to further increase the sequence selectivity and affinity in targeting short RNA, sequences, (such as the nucleotides 2–7, the so-called “seed regions” which are common to several microRNAs)^34^, thus increasing both anti-miR efficiency and diagnostic performances. The design of new modified structures with high affinity and selectivity for these short sequences by trial and error can be time-consuming and labour-intensive, whereas rational design is limited by the scarce availability of crystallized solid-state^35^,^36^,^37^,^38^,^39^,^40^, and NMR resolved structures^41^,^42^.

Numerical simulations based on molecular dynamics (MD) can assist this process allowing for an atomistic understanding of the physics behind the PNA structural organization and enabling a computer-based strategy for *ad hoc* molecular design. In particular, this would enable prediction on PNA molecular activity upon structural modifications and to observe atomistic details of interaction mechanism difficult to be characterized. Such approach allows to selectively choose the most promising PNAs for the synthesis and experimental trials, saving time and resources.

Orozco and coworkers pioneered the exploration of PNA properties employing classical MD techniques. They compared triplex PNA:DNA:PNA structures as reported in literature recording 1.05 ns long trajectories and they found structural parameters in good agreement with experimental ones^43^. Later, they focused on duplex combination PNA:DNA and PNA:RNA concluding that PNA influences duplex structure favoring a P like helix^44^. During the same years Nilsson and co workers proposed molecular modeling and MD simulations on homo- and heteroduplexes involving PNAs and describing base pairing and staking as critical driving-forces of the actual structure^45^,^46^. Gantchev and coworkers investigated *γ*-radiation induced cross-linking in PNA:DNA heteroduplexes by means of a 300 ps long MD, allowing to explain experimental data on cross linking in terms of the effect of terminal capping groups^47^. Hatcher *et al*.^48^ performed studies of electron transfer in PNA and DNA single strands through 2 ns long simulations, highlighting the greater structural fluctuation of the PNA as compared to the DNA moiety and correlating this feature to the observed charge transfer. Mansawat and coworkers, collected 10 ns long MD simulations concerning the use of pyrene moiety as an universal base (i.e. bases able to bind to all four bases) in acpcPNA:DNA duplexes (D-prolyl-2-aminocyclopentane carboxylic acid backbone derivative of PNA), revealing important structural information concerning the spatial arrangement of the inserted modification^49^. Sanders *et al*.^50^ reported 25 ns long simulations on modified PNA bearing a hypoxantine base for the targeting of KRAS2 (i.e. clinically relevant tumor marker) mutated mRNA and they validated their prediction with experimental data. Lastly, Autiero *et al*. studied PNA:DNA and PNA:RNA duplexes focusing on the definition of the most relevant structural parameters^51^,^52^. However, the understanding of the formation mechanism of duplexes made by PNA and RNA (or DNA) and a quantification of the relative stability of modified structures is still missing mainly because of the multitude of variables involved in the hybridization process and by the limited knowledge of the conformational landscape of single-stranded PNA.

In this work, we define a computational strategy based on Well-Tempered Metadynamics (WT-MDMT)^53^,^54^ capable to characterize the flexibility of single stranded PNAs and their degrees of pre-organization in solution. This, helps rationalizing possible hybridization and dissociation mechanism for the PNA case with an approach similar the well-established strategies used for DNA and RNA systems^55^,^56^,^57^,^58^,^59^.

In particular, starting from an experimental PNA:RNA NMR structure (PDB ID: 176D)^41^ we built a simplified PNA single strand model, and investigated its flexibility in solution by means of classical MD and WT-MDMT. Then, we demonstrate how structural modifications of the PNA sequence can induce different degrees of pre-organization affecting significantly the free energy landscape explored by means of WT-MDMT. This finding well compares with the experimental circular dichroism (CD) reported in the literature. The presented Molecular Dynamics and Metadynamics procedure provides two important benefits: it enhance significantly the conformational landscape exploration, and provides details on the formation-dissociation mechanism influencing the PNA:RNA duplex stability; both these can be used in order to finely tune binding selectivity of newly designed PNA structures towards nucleic acids.

We further investigate the stability of the PNA:RNA duplex by simulating dissociation and re-annealing processes. This study revealed the critical importance of the central base pairing of the sequence. This evidence confirms and characterizes the experimental results by Igloi and co workers conducted on mismatch positioning^58^, that highlights greater stability drops for mismatched base in the central part of the PNA compared to those in the terminus. The presented computational approach has two important benefits: it provides details on the formation-dissociation mechanism directly correlated to the PNA:RNA duplex stability, and it defines a predictive methodology for computer screening of a wide scope of modification in order to finely tune the binding selectivity between PNA and nucleic acid strands. Compared to the classical MD simulations applied so far for PNA- related studies, our combined WT-MDMT procedures enhance significantly the conformational landscape exploration potentially allowing for more accurate and reliable prediction on structure-activity correlation.

## Results

**Figure 1:**
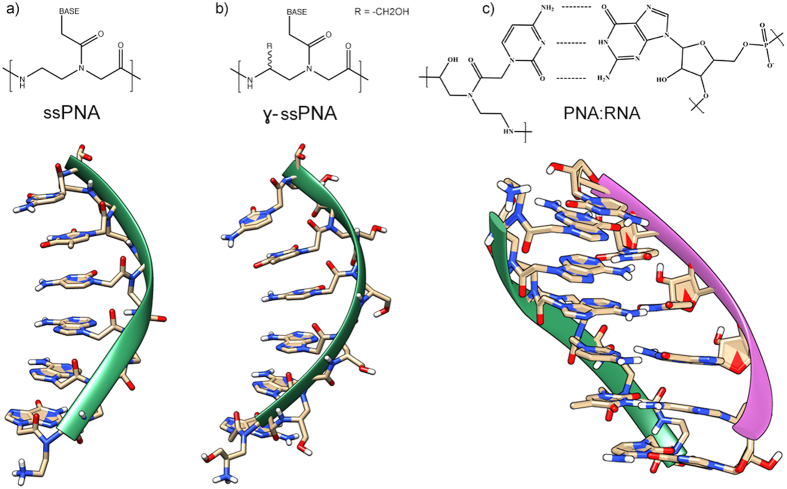
Representation of a generic (**a**) unmodified single stranded PNA (ssPNA), (**b**) *γ* modified PNA(*γ*-ssPNA) and (**c**) duplex PNA:RNA. (PNA: N-term-GAACTC-C-term, RNA 5′-GAGTTC-3′).

In this section we report the most relevant results obtained by combining classical MD and WT-MDMT concerning the conformational freedom of unmodified and *γ*-modified single stranded PNA (i.e. ssPNA and *γ*-ssPNA, Fig. 1a,b respectively) and how it can be correlated to the duplex stability formed by nucleic acid association.

The principal models of gamma-PNAs reported in the literature, were based on serine^29^, alanine^60^,^61^, lysine^31^, or arginine^62^ side chains, and a serine derivative containing mini-PEG chain was more recently introduced for biological applications^63^,^64^. We choose the –*CH*_2_*OH* side group of serine as the simplest polar uncharged residue which can be inserted; since *γ*-PNA with polar groups are very interesting for their improved water- solubility properties, we selected this as a study model, as it can have a more direct impact on biological applications. This type of PNA modification was indeed found to also improve DNA and RNA binding affinity due to a ‘pre-organization’ effect^29^, which was then observed also on other C-5 derivatives^30^. A recent paper also reports the NMR and CD studies on Ser-based *γ*-PNA, showing the existence of marked chiral secondary structures^65^. Thus, as far as the study of overall conformational bias is concerned, the present study can serve to have insights into the properties of other gamma-modified PNAs.

Insights into the key features involved disrupting and assembling PNA:RNA duplexes are also reported by means of thermal induced “melting” and post damage re-annealing.

### Unmodified ssPNA conformations

The initial structure of ssPNA obtained as described in the methodology section, was equilibrated and thermalized at 300 K running a 2 ns long NPT dynamics. Then, a 200 ns productive run was conducted in NVT conditions in order to investigate the conformational freedom of the structure.

Within the first 20 ns no significant modification to the equilibrated system was recorded (Supplementary Information SI1) denoting a certain degrees of stability of the initial helical conformation. Afterwards, critical distortions brought the ssPNA to assume a wide scope of different conformations apparently in equilibrium with each others. However, the initial helical conformation was not observed anymore being statistically infrequent compared to the multitude of other conformations.

We further investigated the complexity of the entire conformational space of ssPNA by means of Metadynamics acting on the collective variables (i.e. Head to Tail distance - HT, and the Stacking Function - Stk) as described in the methodology section. It is worth mentioning that either for the ssPNA and the *γ*-ssPNA the authors tested several collective variables to reach an exhaustive exploration of the associated free energy landscapes. The stacking function turned out to be, by far, the most satisfactory one despite the free energy obtained should be evaluated for their qualitative contents over the quantitative one.

**Figure 2:**
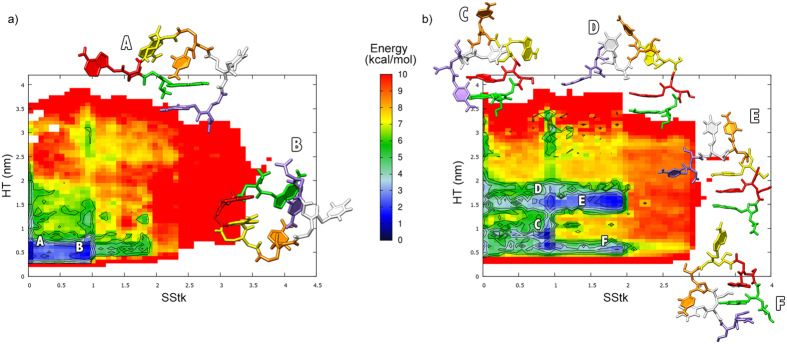
Free energy surface (FES) reconstructed from Gaussian hills deposition of WT-MTMD on (**a**) ssPNA and (**b**) *γ*-ssPNA. Representative structures of principal basins A–F are reported.

In Fig. 2a we report the FES reweighted on the HT and the Sequential Stacking Function SStk according to the scheme proposed by Bonomi *et al*.^66^. (See Supplementary Information SI1b for FES convergence quality) The entire conformational space is covered within 10 kcal/mol and it spans between highly stacked and folded conformations (i.e. large SStk and short HT values) to extremely stretched and unstacked strands (i.e. long HT and small SStk values). The most significant region is the one defined within 2–3 kcal/mol. (i.e. blue wells Fig. 2) The two barrierless minima (A and B) are populated by ssPNA structures characterized by heavily folded and barely stacked conformations.

In particular, the first (i.e. magnification Fig. 2a structure A - see Supplementary Information SI1b uploaded pdb structure A) is characterized by a non-consecutive stacking having place between the first and the last bases of the sequence. (i.e. violet-green) This stacking is not productive for pre-organizing the helical conformation. The second representative structure (i.e. Fig. 2a structure B - see Supplementary Information SI1b uploaded pdb structure B) is barely more organized showing one consecutive stacking. (i.e. red-yellow) However, also this conformation is far from facilitating the hybridization process with DNA and RNA which requires a helical conformation of the PNA.

### *γ*-ssPNA conformations

We employed the same computational approach for the *γ*-modified PNA (*γ*-ssPNA). The starting helical conformation (i.e. after minimization and thermalization) turned to be significantly more persistent than the non modified ssPNA above discussed. The most relevant structural modifications occur only in the latest stage of the unbiased MD run suggesting a remarkable pre-organized behavior. (see Supplementary Information SI2).

Also for this structure we performed WT-MDMT simulations and reweight the FES as described before. In Fig. 2b we report the results of this analysis. (See Supplementary Information SI2b for FES convergence quality) Again, the relevant conformational landscape is explored within 10 kcal/mol and the conformational space explored is equivalent to the one investigated for ss PNA. However, the relative abundance and the statistical relevance of the different conformations are significantly different from the unmodified PNA. The most important region is represented by four well-localized minima (i.e. C–F) all comparable in energy (i.e. <4 kcal/mol) and separated by tiny energy barriers. Beside the structures discovered in C all the other minima do not have any equivalent in the non modified ssPNA. In particular, structures found in D and E are characterized by more stretched conformations (i.e. HT between 1.5–2.0 nm) and sequential stacking significantly higher compared to ssPNA. These structures are mostly important because they share structural parameters comparable to the experimental measurements derived from duplex^41^. Some of the most representative structures localized in the four minima are magnified in Fig. 2C–F. (see Supplementary Information SI2b uploaded pdb structures C-F) Configurations extracted from C and D present the first two bases (Green-Red, Guanine and Adenine) of the sequence in proximal stacking (i.e. distance between the two bases <3 *Å* angstroms). Structures sampled in F and E show several combination of possible stacking between bases. In particular, those sharing a folded configuration (R < 1 nm) are stabilized by two consecutive base stacking (Red-Green, Guanine-Adenine and Orange-White, Cytosine-Thymine Fig. 2) and one further head to tail interaction (Green-Violet, Guanine-Cytosine). The others, characterized by a stretched configuration (E) show consecutive stacking only (i.e. Green-Red, Guanine-Adenine and Red-Yellow, Adenine-Adenine) and hence well-organized towards a possible hybridization with a complementary sequence. The lower degree of freedom for the *γ*-substituted PNA compared with the non-substituted ones is in line with the experimental finding that a smaller entropy loss is observed upon hybridization with DNA and RNA, even though this “preorganization” is not due to a perfectly helical single strand structure. Actually, the flexibility of these molecules is still very high. (see Supplementary Information SI2b 10 ns movie extracted from the original 500 ns long WT-MDMT trajectory).

### PNA:RNA interactions

**Figure 3:**
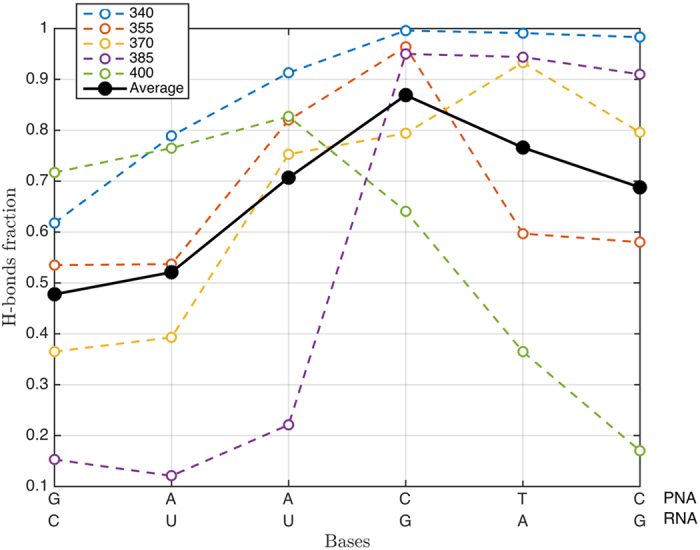
Contribution to the H-bonding of each couple of bases: this parameter is calculated as the average H-Bonding, calculated with the CV called “Coordination” as implemented in PLUMED2 and described in details in the manual^71^, before duplex disruption. PNA sequence is written from N-terminus to C-terminus, RNA sequence is written from 3′ to 5′.

The dissociation process of a pre-formed duplex is the most direct and intuitive analysis in order to understand the atomistic details of the interaction between PNA with complementary nucleic acids. We performed several MD simulations on the same PNA:RNA duplex (Fig. 1c), at different temperatures (i.e. between 340 K–400 K) inducing complete dissociation. In order to quantify the contribution of each base pairing to the overall stability of the duplex we defined the single-base H-bonding fraction calculated 0.5 ns before the complete duplex disruption. (see Supplementary Information SI3c for details) In Fig. 3 we report this quantity along with the base pairing sequence (N-term to C-term for PNA and 3′ → 5′ for RNA) at the different temperatures (i.e. dashed curves). The average trend reported (i.e. solid black demonstrates that in the range of temperature analyzed, the duplex dissociation occurs preserving the central base pairing until complete degradation. Interestingly, the duplex segment containing the C-terminal part of the PNA (CTC) section of the PNA (i.e. right branch of the plot reported in Fig. 3) is significantly more persistent than that at N-terminal PNA segment (GAA) suggesting a marked sequence-dependent effect on the thermal stability of the duplex. However, this behavior is not strictly respected as demonstrated by the H-bonding fraction calculated at 400 K remarking the importance of having a statistically representative pool of simulations.

**Figure 4:**
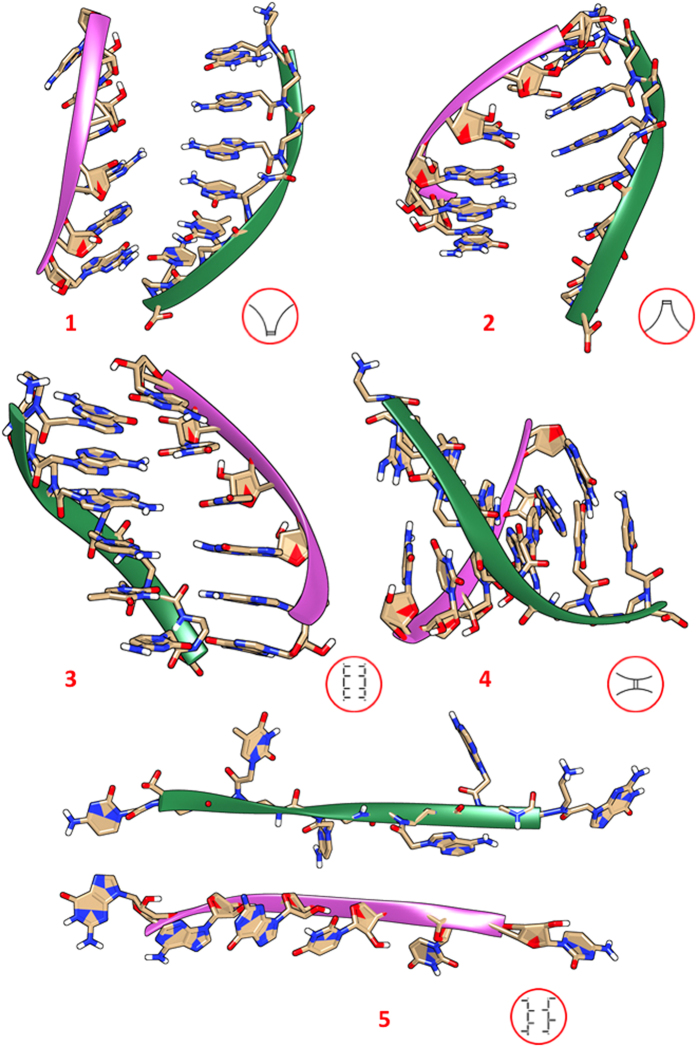
Distorted duplex structures: (1) spread like a fan on C:5′ end; (2) spread like a fan on N:3′ end; (2) strands shifted of 5–6 *Å*; (4) twisted strands (5) stretched strands. The symbolic representations of stress induced in the system are represented in red circles. In Supplementary Information SI3 we report the pairing behaviors for all cases including structure **3** that is the only one capable to stably converge into the duplex in less than 5 ns.

In order to understand possible mechanisms that lead to the formation/dissociation of PNA:RNA duplex, we applied our methodology to the study of double strand re-annealing after an induced external stress. The size of the re-annealing conformational space is enlarged by an enormous amount of degrees of freedom and did not allow to converge WT-MDMT free energies. For this reason we employed standard molecular dynamics starting from several different geometries (Fig. 4) selected from the thermally-induced dissociation trajectories above discussed. This strategy enforces the statistical meaning of the MD trajectories and allows driving qualitative conclusions concerning the reassociation mechanism. In particular we selected different geometries nearby the thermally-induced dissociation that can be clustered in four main categories: 1–2) head or tail linked, 3) homogeneously stretched H-bonding, and 4) X-twisted respect to the center of the duplex. In particular, structures **1** and **2** are spread like a fan on 5′ and 3′ ends, whereas **3** has been selected from an intermediate configuration characterized by an average distance between the two strands of 5–6 *Å*, and in **4** the two strands are twisted. In order to increase the statistical significance of our starting pool of configurations we extracted a rarely populated fully-stretched configuration from the WT-MTMD conducted on the ssPNA (Fig. 2) and performed similar calculations on the RNA single strand. Then, we progressively approached the two single strands and minimized the geometry obtaining the starting point for re-annealing **5**.

For each system we performed a 100 ns long MD simulation and we tracked the number of bases pairing as an index of duplex formation or disruption. This parameter is defined as the fraction of the persistent H-bonding between base paired respect to those generated by a complete duplex formation. Among the different scenarios, (see Supplementary Information SI3) only **3** reaches complete base pairing in the early stage of the simulation leading to a well-stabilized duplex in less then 5 ns. As expected, a preorganized orientation of the two strands drives the re-annealing process facilitating the binding process. Satisfactory is also the re-annealing observed for **4** where the two strands can initially interact through the central base pairing. In this case, the re-orientation of the two units is entropically more demanding and longer time scales for reorganization are necessary. Systems **1** and **2** are initially annealed by one single base pairing at the PNA C-terminus and N-terminus position respectively. However, the former does not evolve toward any grade of annealing whereas the latter slowely reorganizes in a double helix within 60–70 ns. The different behavior recorded for **1** and **2** demonstrate that non-symmetric sequence could potentially favor the re-annealing in one single direction. This evidence further supports our previous finding relatively to the sequence-dependent behavior in the thermal dissociative process. Lastly, a disordered mismatching between the two fragments as set in **5** leads to complete failure in the re-annealing process. From this analysis we conclude that the pre-organization of the two strands is of critical importance in facilitating the duplex formation and that the interaction between the bases placed in the central part of the sequence presumably play a critical role in this process.

## Discussion

Understanding single strand degrees of freedom is of paramount importance in order to predict the specific capability of PNA structures to interact, and eventually bind, RNA. In particular, the backbone flexibility, functionalization with *ad hoc* moieties and the base sequence play a critical role in pre-organizing the single strand and successively binding the desired target. Our MD and WT-MDMT simulations suggested that the unmodified ssPNA considered does not assume conformations that would facilitate the hybridization process with nucleic acids. Moreover, we concluded that short MD simulations (i.e. in our case shorter than 20 ns) would drive to misleading conclusions being the ssPNA helical structure relatively stable for shorter timeframes. On the contrary, MD and WT-MDMT runs conducted on the *γ*-ssPNA structure demonstrated a remarkable stability of the pre-organized helical conformation imputable to the –*CH*_2_*OH* functionalization placed in *γ*. This finding is in agreement with earlier experimental observation proposed by Ly and co-workers^29^. Our Metadynamics simulations conducted on single strand PNAs show a delicate balance between enthalpy contribution generated by intra-strand base stacking and entropy effects, which lead to the chaotic folding of the sequence. These two contributions respectively favor and disfavor a helical preorganization that would facilitate PNA:RNA interaction. The unmodified ssPNA (Fig. 1a) and gamma modified *γ*-ssPNA (Fig. 1b) show substantially different free energy landscape with respect to their conformational freedom. In particular, ssPNA assumes preferably folded conformations characterized by low sequential stacking between the basis.

The most representative structures extracted from minima A and B (Fig. 2a) are dramatically different from the one that should be assumed in the PNA:RNA duplex. Conversely, *γ*-ssPNA demonstrates a higher level of pre-organization generating stable conformations with suitable combinations of sequential base stacking and fragment folding to generate helical conformations. A recent paper also suggested the occurrence of a chiral preferred secondary structure^65^. Structures extracted from basin E (Fig. 2b) are of particular interest being characterized by the preservation of structural properties founded experimentally in the duplex. In particular, these structures have an optimal HT length (i.e. 2.0–2.2 nm) and show sequential stacking only. Hence, such conformations are well organized towards a possible hybridization with a complementary sequence. For this reason, structures from basin E are the most relevant supporting our thesis that mostly ascribe to functionalization in gamma position of the PNA backbone the opportunity to finely tune the binding affinity of nucleic acid moieties.

Structural analysis on the structures C-F of Fig. 2 showed that the torsional angle formed by the *H*–*N*–*C*–*H* adjacent to the amide bond is substantially planar (−173.0° ± 21.4°) allowing the –*CH*_2_*OH* occupying non hindered regions and favoring several polar interactions with the surrounding solvent. Unlike these, structures A and B of unmodified ssPNA showed, for *H*–*N*–*C*–*H* with the C-H bond of equivalent stereochemical position (pro-R hydrogen), a different value and wider range (−90.7° ± 76.3°). Moreover, the hydrogen-hydrogen average distance between sequential amino acids (see Supplementary Information SI2c) in the *γ*-modified falls in a very narrow range compared to the one measured for the ssPNA. These findings, confirm our hypothesis denoting the considerably higher flexibility of ssPNA and the higher helical propensity of *γ*-ssPNA. It is expected that properly preorganized *γ*-ssPNA strands would require significantly less free energy to associate in the duplex compared to the unmodified ssPNA as they require a smaller entropy component.

These results are compatible with Circular Dichroism (CD) experiments conducted on similar PNA^29^ structures suggesting that backbone functionalization can limit the single strand flexibility and favoring either total or partial helical conformations as revealed in the CD spectra. Such a reduced backbone flexibility favors the hybridization process, as supported by the melting temperature (*T*_*m*_) always higher for the gamma modified PNA and enforces the view that entropic contribution are decisive for this enhanced affinity. Most importantly, the present results allow to enlarge the view of this type of “preorganization” and assess more precisely what can be expected from this type of modified backbones; in fact, the presence of the *γ*-side chains determines a local helical arrangement, and not a entirely helical single-stranded structure^29^,^67^.

The dissociation study, on the other hand, allowed to understand the role of the single bases and of their positioning in the stabilization of the PNA:RNA duplex. It was reported that structural variations affect the stability of PNA:DNA duplexes mainly by slower dissociation processes, while that of association is much less affected^63^. By increasing the temperature, the kinetic energy of the system grows along with the probability for base un-pairing. This induces the duplex to dissociate due to the loss of hydrogen bonds and the increasing of entropy. Before duplex complete disruption we can analyze which couples of bases contributes more to H-bonding. Simulated data revealed that averagely, pairing among central bases resulted more persistent compared to those of terminal bases (Fig. 3). The asymmetric shape of the curve is probably due to the specific sequence considered (GAACTC): the first half of the duplex presents a total of 7 hydrogen bonds while the second half 8, slightly favoring the C-terminal compared to the N-terminal. These results further suggest the importance of central bases in both formation and dissociation process. A direct consequence of this finding is that in order to improve PNA binding properties, functional modifications must be focused on stabilizing central part of the PNA. The further effect of introducing modifications in different position of the sequence has not been specifically studied, but can be deduced by many experimental data^68^,^69^.

Re-annealing MD simulations starting from different damaged PNA:RNA complexes highlight some critical aspects that need to be considered for functional sequence design. Structures **1**–**5** in Fig. 4 sample some of the possible scenarios that can be derived during either the PNA:RNA formation or dissociation process that can be tracked by bases pairing as discussed in the previous section and reported in Supplementary Information SI3. As expected the time estimated for a complete or partial re-annealing depends on the distortion initially induced on the duplex. Among the five different scenarios, structure **3** reaches an optimal and complete base pairing within the nanoseconds time scale. In this case the re-annealing is particularly favored having the complementary bases of the two strands properly oriented each other. The re-annealing is limited by diffusion of the two strands originally placed at ~6 *Å* and the driving force of this process are long-range Van der Waals interactions. Structures **1** and **2** (Fig. 4) describe two similar scenarios being characterized by one residual base pairing in C- and N- terminus respectively. For palindromic DNA:DNA or RNA:RNA sequences the two structures would be equivalent. However, this is not the case of our PNA and the dynamical evolution of the two systems is substantially different showing a higher attitude to re-annealing for structure **2**. This finding, suggests that rational design of the PNA sequence should be accurately considered to drive the association process. Another important aspect is the base interaction localized in the central part of the sequence. Our simulations show that a preformed base pairing, as the one characterizing structure **4**, (Fig. 4) promotes and assists the formation of the other proximal pairing. Differently from simulations of **2** and **3** where the re-annealing process is substantially one-way direction, in **4** we observe the simultaneous re-annealing on the two branches starting from the central base pair. The time needed for duplex formation is shorter than 10 ns and the mechanism is driven by inter H-bonding formation and intra base stacking. This finding is supported by the single-contribution analysis performed on **2**, **3** and **4** simulations where we discovered that pairing central-sequence bases trigger the overall association process more efficiently than any other base pairing (see Supplementary Information SI3b). Lastly, unphysical scenarios as the one represented in structure **5** remarks how critical is the pre-organization of PNA in promoting the hybridization process. As demonstrated by **5** dynamics (see Supplementary Information SI3a) the two fully strained PNA and RNA fragments with randomly oriented bases cannot generate any paring and eventually lose any interaction favoring single strand folding. The interaction models considered in these simulations are not certainly sufficient to describe all possible ways of contact between the two strands. However, this study demonstrates that the orientation of the interface between bases are crucial; central bases exhibited unique properties in favoring duplex formation, suggesting a possible mechanism of formation of the duplex starting from this position.

## Methods

All the MD simulations reported in this work were carried out with GROMACS 4.5^70^, patched with PLUMED 2.0^71^, for the Metadynamics calculations. The 6 mer structure 176D (Supplementary Information SI1, PNA sequence: H-GAACTC-OH)^41^, taken from Protein Data Bank, was chosen as starting structure for the computational studies on unmodified PNAs complexes with RNA since it was one of the few PNA:RNA duplex structures reported in literature. Longer 8 mer PNA:RNA duplex has been resolved recently by Kiliszek and co workers^72^ and to the best of our knowledge that is the only case reported in literature. However, being the RNA “core” seed region discussed above made by a 6 mer sequence and being interested in its targeting, we focused on the 176D derivatives only. The original structure was solvated with explicit TIP3P^73^ water molecules, in a cubic box of 41.3 × 50.4 × 45.2 *Å*^3^ with five atoms of sodium added as counterions. The final system is made by about 9400 atoms (see Supplementary Information SI4). As no crystal structure of single strand PNA (i.e. in the text ssPNA) (Fig. 1) is available in literature we created a simplified model by removing the RNA strand from the original NMR experimental structure. This is an approximation of the stable ssPNA structure, but it represents the only good starting point for further analysis. The system is expected to be much more flexible and disordered than the parent duplex and for this reason we avoided periodic boundary interaction between images by solvating in a larger 70.7 × 70.9 × 71.0 *Å*^3^ cubic box. The final system totally counts, including totally about 34700 atoms. The *γ*-modified PNA structures (i.e. both duplex and single strand) were generated by insertion of serine groups in gamma position of the PNA moiety of 176D. Duplex was solvated with TIP3P water molecules in a 58.9 × 55.1 × 57.5 *Å*^3^ cubic box, counting in total about 18300 atoms (see Supplementary Information SI5), while single stranded PNA (i.e. in the text *γ*-ssPNA) (Fig. 1) was solvated in a 70.7 × 70.7 × 70.7 *Å*^3^ cubic box for a total of about 34700 atoms. For the duplex re-annealing and dissociation studies we considered the structure 176D opportunely modified in order to simulate structural strains. Systems were solvated in cubic boxes filled with TIP3P water molecules (box size and atoms: structure **1** 45.3 × 52.9 × 52.0 *Å*^3^, 12300 atoms; structure **2** 53.4 × 54.2 × 55.0 *Å*^3^, 11900; structure **3** 48.9 × 58.5 × 47.0 *Å*^3^, 10000; structure **4** 51.6 × 54.2 × 50.6 *Å*^3^, 10500; structure **5** 57.6 × 53.0 × 75.0 *Å*^3^, 17800).

For simulations where RNA moieties were present, we employed ff99SB^74^ force field (FF) improved with parmbsc0^75^, since these force fields have been extensively used leading to reliable and reproducible results^76^,^77^. PNAs are not parameterized in the available FFs and we retrieved missing parameters from R.E.DD.B. Server^78^,^79^ obtained according to the scheme proposed by Cornell *et al*.^80^. Similarly, a force field for gamma serine modified PNAs is not available in the literature and its derivation was conducted by us following the same protocol used for unmodified PNA. For the validation and description of the employed FF see Supplementary Information SI6. The exhaustive conformational characterization of the ssPNA was enhanced by the Metadynamics algorithm. In particular, we employed two Collective Variables (CVs): the monomers head-to-tail center of mass distance (HT) of the single strands, which distinguish between “folded” and “unfolded” PNA structures and, the stacking function (Stk) describing the grade of interaction between different bases. The latter, is a linear combination of functions built for describing the local order between two or more nucleobases evaluating orientation and distance of the aromatic units^81^. More details about the Stk collective variable are reported in the Supplementary Information (SI7). To build the Metadynamics bias during the simulation we deposited every picoseconds a Gaussian hill with width of 0.01 nm for HT and 0.01 on Stk and height of 2.5 kJ. To ensure the convergence of the Free-energy calculation, we adopted the Well-Tempered Metadynamics scheme (WT-MDMT)^54^ with gamma equal to 30. For ssPNA and for *γ*-ssPNA we carried out 400 ns long WT-MDMTs, and the simulations were stopped after verifying that the local minima converged. For discriminating among all possible conformations explored by WT-MDMT we post processed (i.e. reweighting scheme)^66^ the collected data by means of two different collective variables jointly to HT. In particular, these two CVs distinguish the stacking interaction between sequential bases (SStk) and non-sequential ones. In order to study single strand behavior in relation to the duplex is necessary to decompose the two contributions in respect of the total stacking. In particular, conformations with preferred sequential stacking are more likely to assume duplex disposition, while structures with non-sequential stacking are far away from desired duplex conformation. All MD simulations started from minimized conformations successively equilibrated at the desired temperature and pressure equal to 1 atm. The equilibration process last for 2 ns in the NPT ensemble, employing the Berendsen barostat^82^. The production runs were generated in the NVT ensemble, by means of the v-rescale thermostat^83^. The non-bonded cutoff scheme was set equal to 10 *Å* and the PME algorithm was employed for the electrostatic contribution. The simulations were performed with a 2.0 fs time step, constraining all the bonds with the LINCS algorithm^84^.

## Additional Information

**How to cite this article**: Verona, M. D. *et al*. Focus on PNA Flexibility and RNA Binding using Molecular Dynamics and Metadynamics. *Sci. Rep.* **7**, 42799; doi: 10.1038/srep42799 (2017).

**Publisher's note:** Springer Nature remains neutral with regard to jurisdictional claims in published maps and institutional affiliations.

## Supplementary Material

Supplementary Information

Supplementary Video S1

Supplementary Video S2

## References

[b1] Nielsen P., Egholm M., Berg R. & Buchardt O. Sequence-selective recognition of DNA by strand displacement with a thymine-substituted polyamide. Science 254, 1497–1500 (1991). 1962210 10.1126/science.1962210

[b2] Nielsen P. & Appella D. Peptide Nucleic Acids Methods and Protocols, 2nd edn. (Humana Press, Springer Science Business Media, 2014).

[b3] Nielsen P. E. *PNA* technology. Mol. Biotechnol. 26, 233–248 (2004). 15004293 10.1385/MB:26:3:233

[b4] Bertucci A., Manicardi A. & Corradini R. Advanced Molecular Probes for Sequence-Specific DNA Recognition. Detection of non-amplified genomic DNA (Springer Science & Business Media, 2012).

[b5] Brind’Amour J. & Lansdorp P. Analysis of repetitive *PNA* in chromosomes by flow cytometry. Nat. Meth. 8, 484–486 (2011). 10.1038/nmeth.1601 PMC3753228 21532581

[b6] Hu J. & Corey D. R. Inhibiting gene expression with peptide nucleic acid *PNA* peptide conjugates that target chromosomal DNA. Biochemistry 46, 7581–7589 (2007). 17536840 10.1021/bi700230a PMC2564818

[b7] Cogoi S. . Transcription inhibition of oncogenic *KRAS* by a mutation-selective peptide nucleic acid conjugated to the *PKKKRKV* nuclear localization signal peptide. Biochemistry 44, 10510–10519 (2005). 16060660 10.1021/bi0505215

[b8] Tonelli R. . Antitumor activity of sustained *N*-myc reduction in rhabdomyosarcomas and transcriptional block by antigene therapy. Clin. Cancer Res. 18, 796–807 (2012). 22065083 10.1158/1078-0432.CCR-11-1981

[b9] Nielsen P. E. Peptide nucleic acids (*PNA*) in chemical biology and drug discovery. Chem. Biodivers. 7, 786–804 (2010). 20397216 10.1002/cbdv.201000005

[b10] Kaihatsu K., Huffman K. E. & Corey D. R. Intracellular uptake and inhibition of gene expression by *PNA*s and *PNA*-peptide conjugates. Biochemistry 43, 14340–14347 (2004). 15533038 10.1021/bi048519l

[b11] Liu Y., Braasch D. A., Nulf C. J. & Corey D. R. Effand isoform-selective inhibition of cellular gene expression by peptide nucleic acids. Biochemistry 43, 1921–1927 (2004). 14967032 10.1021/bi0358519

[b12] Turner J. J. . *RNA* targeting with peptide conjugates of oligonucleotides, si*RNA* and *PNA*. Blood Cells, Mol. Dis. 38, 1–7 (2007). 17113327 10.1016/j.bcmd.2006.10.003

[b13] Kayali R., Bury F., Ballard M. & Bertoni C. Site-directed gene repair of the dystrophin gene mediated by *PNA*–sso*DN*s. Hum. Mol. Genet. 19, 3266–3281 (2010). 20542988 10.1093/hmg/ddq235

[b14] Di Leva G., Garofalo M. & Croce C. M. Micro*RNA*s in cancer. Annu. Rev. Pathol. Mech. Dis. 9, 287–314 (2014). 10.1146/annurev-pathol-012513-104715 PMC4009396 24079833

[b15] Van Rooij E. & Kauppinen S. Development of micro*RNA* therapeutics is coming of age. EMBO Mol. Med. 851–864 (2014). 24935956 10.15252/emmm.201100899 PMC4119351

[b16] Fabani M. M. & Gait M. J. mir-122 targeting with *LNA*/29-o-methyl oligonucleotide mixmers, peptide nucleic acids *PNA*, and *PNA* peptide conjugates. RNA 14, 336–346 (2008). 18073344 10.1261/rna.844108 PMC2212241

[b17] Gambari R. . Targeting micro*RNAs* involved in human diseases: a novel approach for modification of gene expression and drug development. Biochem. Pharmacol. 82, 1416–1429 (2011). 21864506 10.1016/j.bcp.2011.08.007

[b18] Calin G. A. & Croce C. M. Micro*RNA*-cancer connection: The beginning of a new tale. Cancer Res. 66, 7390–7394 (2006). 16885332 10.1158/0008-5472.CAN-06-0800

[b19] Volinia S. . A micro*RNA* expression signature of human solid tumors defines cancer gene targets. Proc. Natl. Acad. Sci. USA 103, 2257–2261 (2006). 16461460 10.1073/pnas.0510565103 PMC1413718

[b20] Fabbri E. . Modulation of the biological activity of micro*RNA*-210 with peptide nucleic acids (*PNA*s). ChemMed-Chem 6, 2192–2202 (2011). 10.1002/cmdc.201100270 22012891

[b21] Oh S. Y., Ju Y. & Park H. A highly effective and long-lasting inhibition of mi*RNA*s with *PNA*-based antisense oligonucleotides. Mol. Cells 28, 341–345 (2009). 19812898 10.1007/s10059-009-0134-8

[b22] Fabani M. M. . Efficient inhibition of mir-155 function in vivo by peptide nucleic acids. Nucleic Acids Res. 4466–4475 (2010). 20223773 10.1093/nar/gkq160 PMC2910044

[b23] Bertucci A. . Combined delivery of temozolomide and anti-mir-221 *PNA* using mesoporous silica nanoparticles induces apoptosis in resistant glioma cells. Small 11, 5687–5695 (2015). 26395266 10.1002/smll.201500540

[b24] Ryoo S.-R. . Quantitative and multiplexed micro*RNA* sensing in living cells based on peptide nucleic acid and nano graphene oxide (*PANGO*). ACS nano 7, 5882–5891 (2013). 23767402 10.1021/nn401183s

[b25] Corradini R. . Peptide nucleic acids with a structurally biased backbone. updated review and emerging challenges. Curr. Top. Med. Chem. 11, 1535–1554 (2011). 21510833 10.2174/156802611795860979

[b26] Sugiyama T. & Kittaka A. Chiral peptide nucleic acids with a substituent in the *N*-(2-aminoethy)glycine backbone. Molecules 18, 287–310 (2012). 23271467 10.3390/molecules18010287 PMC6269907

[b27] Wojciechowski F., Hudson E. & Robert H. Nucleobase modifications in peptide nucleic acids. Current Topics in Medicinal Chemistry 7, 667–679 (2007). 17430208 10.2174/156802607780487795

[b28] Sforza S., Tedeschi T., Corradini R. & Marchelli R. Induction of helical handedness and dna binding properties of peptide nucleic acids (*PNA*s) with two stereogenic centres. European J. Org. Chem. 2007, 5879–5885 (2007).

[b29] Dragulescu-Andrasi A. . A simple *γ*-backbone modification preorganizes peptide nucleic acid into a helical structure. J. Am. Chem. Soc. 128, 10258–10267 (2006). 16881656 10.1021/ja0625576

[b30] Crawford M. J., Rapireddy S., Bahal R., Sacui I. & Ly D. H. Effect of steric constraint at the *γ*-backbone position on the conformations and hybridization properties of *PNA*s. J. Nucleic Acids 2011, 652702 (2011). 21776375 10.4061/2011/652702 PMC3138043

[b31] Englund E. A. & Appella D. H. *γ*-substituted peptide nucleic acids constructed from l-lysine are a versatile scaffold for multifunctional display. Angew. Chemie - Int. Ed. 46, 1414–1418 (2007). 10.1002/anie.200603483 17133633

[b32] De A., Souchelnytskyi S., van den Berg A. & Carlen E. T. Peptide nucleic acid (*PNA*)–*DNA* duplexes: Comparison of hybridization affinity between vertically and horizontally tethered *PNA* probes. ACS Appl. Mater. Interfaces 5, 4607–4612 (2013). 23668364 10.1021/am4011429

[b33] Rapireddy S., He G., Roy S., Armitage B. A. & Ly D. H. Strand invasion of mixed-sequence *B*–*DNA* by acridine-linked, *γ*-peptide nucleic acid (*γ*-pna). J. Am. Chem. Soc. 129, 15596–15600 (2007). 18027941 10.1021/ja074886j

[b34] Obad S. . Silencing of micro*RNA* families by seed-targeting tiny lnas. Nat. Genet. 43, 371–378 (2011). 21423181 10.1038/ng.786 PMC3541685

[b35] Menchise V. . Insights into peptide nucleic acid (*PNA*) structural features: The crystal structure of a *D*-lysine-based chiral *PNA-DNA* duplex. Proc. Natl. Acad. Sci. USA 100, 12021–12026 (2003). 14512516 10.1073/pnas.2034746100 PMC218706

[b36] He W. . The structure of a *γ*-modified peptide nucleic acid duplex. Mol. BioSyst. 6, 1619–1629 (2010). 20386807 10.1039/c002254c

[b37] Petersson B. . Crystal structure of a partly self-complementary peptide nucleic acid (*PNA*) oligomer showing a duplex-triplex network. J. Am. Chem. Soc. 127, 1424–1430 (2005). 15686374 10.1021/ja0458726

[b38] Betts L., Josey J. A., Veal J. M. & Jordan S. R. A nucleic acid triple helix formed by a peptide nucleic acid-*DNA* complex. Science 270, 1838–1841 (1995). 8525381 10.1126/science.270.5243.1838

[b39] Rasmussen H., Kastrup J. S., Nielsen J. N., Nielsen J. M. & Nielsen P. E. Crystal structure of a peptide nucleic acid (*PNA*) duplex at 1.7 a resolution. Nat. Struct. Biol. 4, 98–101 (1997). 9033585 10.1038/nsb0297-98

[b40] Yeh J. I. . Crystal structure of chiral *γPNA* with complementary dna strand: Insights into the stability and specificity of recognition and conformational preorganization. J. Am. Chem. Soc. 132, 10717–10727 (2010). 20681704 10.1021/ja907225d PMC2929025

[b41] Brown S. C., Thomson S. A., Veal J. M., Davis D. G. . *NMR* solution structure of a peptide nucleic acid complexed with *RNA*. Science 265, 777–780 (1994). 7519361 10.1126/science.7519361

[b42] Eriksson M. & Nielsen P. E. Solution structure of a peptide nucleic acid–*DNA* duplex. Nat. Struct. Mol. Biol. 3, 410–413 (1996). 10.1038/nsb0596-410 8612069

[b43] Shields G. C., Laughton C. A. & Orozco M. Molecular dynamics simulation of a *PNA:DNA:PNA* triple helix in aqueous solution. J. Am. Chem. Soc. 120, 5895–5904 (1998).

[b44] Soliva R., Sherer E., Luque F. J., Laughton C. A. & Orozco M. Molecular dynamics simulations of *PNA:DNA* and *PNA:RNA* duplexes in aqueous solution. J. Am. Chem. Soc. 122, 5997–6008 (2000).

[b45] Sen S. & Nilsson L. Molecular dynamics of duplex systems involving *PNA*: Structural and dynamical consequences of the nucleic acid backbone. Journal of the American Chemical Society 120, 619–631 (1998).

[b46] Sen S. & Nilsson L. Md simulations of homomorphous *PNA, DNA*, and *RNA* single strand: Characterization and comparison of conformations and dynamics. Journal of the American Chemical Society 123, 7414–7422 (2001). 11472173 10.1021/ja0032632

[b47] Gantchev T. G. .*γ*-radiation induced interstrand cross-links in *PNA:DNA* heteroduplexes. Biochemistry 48, 7032–7044 (2009). 19469551 10.1021/bi9002474

[b48] Hatcher E., Balaeff A., Keinan S., Venkatramani R. & Beratan D. N. *PNA* versus *DNA*: effects of structural fluctuations on electronic structure and hole-transport mechanisms. J. Am. Chem. Soc. 130, 11752–11761 (2008). 18693722 10.1021/ja802541e

[b49] Mansawat W., Boonlua C., Siriwong K. & Vilaivan T. Clicked polycyclic aromatic hydrocarbon as a hybridization-responsive fluorescent artificial nucleobase in pyrrolidinyl peptide nucleic acids. Tetrahedron 68, 3988–3995 (2012).

[b50] Sanders J. M. . Effects of hypoxanthine substitution in peptide nucleic acids targeting *KRAS*2 oncogenic m*RNA* molecules: theory and experiment. J. Phys. Chem. B 117, 11584–11595 (2013). 23972113 10.1021/jp4064966 PMC3946533

[b51] Autiero I., Saviano M. & Langella E. Conformational studies of chiral *D*-lys-*PNA* and achiral *PNA* system in binding with *DNA* or *RNA* through a molecular dynamics approach. Eur. J. Med. Chem. 91, 109–117 (2015). 25112690 10.1016/j.ejmech.2014.08.015

[b52] Autiero I., Saviano M. & Langella E. Molecular dynamics simulations of *PNA-PNA* and *PNA-DNA* duplexes by the use of new parameters implemented in the *GROMACS* package: a conformational and dynamics study. Phys. Chem. Chem. Phys. 16, 1868–1874 (2014). 24327011 10.1039/c3cp54284j

[b53] Laio A. & Parrinello M. Escaping free-energy minima. Proc. Natl. Acad. Sci. USA 99, 12562–12566 (2002). 12271136 10.1073/pnas.202427399 PMC130499

[b54] Barducci A., Bussi G. & Parrinello M. Well-tempered metadynamics: a smoothly converging and tunable free-energy method. Phys. Rev. Lett. 100, 020603 (2008). 18232845 10.1103/PhysRevLett.100.020603

[b55] Maciejczyk M., Spasic A., Liwo A. & Scheraga H. A. Dna duplex formation with a coarse-grained model. J. Chem. Theory Comput. 10, 5020–5035 (2014). 25400520 10.1021/ct4006689 PMC4230386

[b56] Špacková N. . Molecular dynamics simulations and thermodynamics analysis of *DNA*-drug complexes. minor groove binding between 4’, 6-diamidino-2-phenylindole and dna duplexes in solution. J. Am. Chem. Soc. 125, 1759–1769 (2003). 12580601 10.1021/ja025660d

[b57] Cheatham T. E. & Kollman P. A. Molecular dynamics simulations highlight the structural differences among *DNA: DNA, RNA: RNA*, and *DNA: RNA* hybrid duplexes. J. Am. Chem. Soc. 119, 4805–4825 (1997).

[b58] Igloi G. L. Variability in the stability of *DNA*–peptide nucleic acid (*PNA*) single-base mismatched duplexes: Real-time hybridization during affinity electrophoresis in *PNA*-containing gels. Proc. Natl. Acad. Sci. USA 95, 8562–8567 (1998). 9671717 10.1073/pnas.95.15.8562 PMC21115

[b59] Limongelli V. . The *γ*-triplex dna. Angew. Chemie - Int. Ed. 52, 2269–2273 (2013). 10.1002/anie.201206522 23335456

[b60] Rapireddy S., He G., Roy S., Armitage B. A. & Ly D. H. Strand invasion of mixed-sequence B-DNA by acridine-linked, γ-peptide nucleic acid (γ-PNA). J. Am. Chem. Soc. 129, 15596–15600 (2007). 18027941 10.1021/ja074886j

[b61] Wierzbinski E. . Effect of backbone flexibility on charge transfer rates in peptide nucleic acid duplexes. Journal of the American Chemical Society 134, 9335–9342, PMID: 22548314 (2012). 22548314 10.1021/ja301677z

[b62] Manicardi A. . Cellular uptakes, biostabilities and anti-mir-210 activities of chiral arginine-pnas in leukaemic k562 cells. ChemBioChem 13, 1327–1337 (2012). 22639449 10.1002/cbic.201100745 PMC3401907

[b63] Sahu B. . Synthesis and characterization of conformationally preorganized, (r)-diethylene glycol-containing *γ*-peptide nucleic acids with superior hybridization properties and water solubility. The Journal of Organic Chemistry 76, 5614–5627, PMID: 21619025 (2011). 21619025 10.1021/jo200482d PMC3175361

[b64] Dutta S., Armitage B. A. & Lyubchenko Y. L. Probing of minipeg*γ*-pna–dna hybrid duplex stability with afm force spectroscopy. Biochemistry 55, 1523–1528, PMID: 26898903 (2016). 26898903 10.1021/acs.biochem.5b01250 PMC4792705

[b65] Viéville J., Barluenga S., Winssinger N. & Delsuc M. Duplex formation and secondary structure of *γ*-pna observed by {NMR} and {CD}. Biophysical Chemistry 210, 9–13, Special Issue on Chemical Complexity and Biology (2016). 26493008 10.1016/j.bpc.2015.09.002

[b66] Bonomi M., Barducci A. & Parrinello M. Reconstructing the equilibrium *B*oltzmann distribution from well-tempered metadynamics. J. Comput. Chem. 30, 1615–1621 (2009). 19421997 10.1002/jcc.21305

[b67] Manna A., Rapireddy S., Sureshkumar G. & Ly D. H. Synthesis of optically pure *γ*-PNA monomers: a comparative study. Tetrahedron 71, 3507–3514 (2015). 10.1016/j.tet.2015.03.052 PMC6379906 30792557

[b68] Sforza S., Haaima G., Marchelli R. & Nielsen P. E. Chiral peptide nucleic acids (*PNA*s): helix handedness and *DNA* recognition. European J. Org. Chem. 1999, 197–204 (1999).

[b69] Sen A. & Nielsen P. E. Hydrogen bonding versus stacking stabilization by modified nucleobases incorporated in *PNA: DNA* duplexes. Biophys. Chem. 141, 29–33 (2009). 19162391 10.1016/j.bpc.2008.12.006

[b70] Pronk S. . Gromacs 4.5: a high-throughput and highly parallel open source molecular simulation toolkit. Bioinformatics btt055 (2013). 10.1093/bioinformatics/btt055 PMC3605599 23407358

[b71] Tribello G. A., Bonomi M., Branduardi D., Camilloni C. & Bussi G. Plumed 2: New feathers for an old bird. Comput. Phys. Commun. 185, 604–613 (2014).

[b72] Kiliszek A., Banaszak K., Dauter Z. & Rypniewski W. The first crystal structures of *RNA-PNA* duplexes and a *PNA-PNA* duplex containing mismatchesÑtoward anti-sense therapy against treds. Nucleic Acids Res. gkv1513 (2015). 10.1093/nar/gkv1513 PMC4770230 26717983

[b73] Jorgensen W. L., Chandrasekhar J., Madura J. D., Impey R. W. & Klein M. L. Comparison of simple potential functions for simulating liquid water. The Journal of Chemical Physics 79 (1983).

[b74] Lindorff-Larsen K. . Improved side-chain torsion potentials for the amber ff99sb protein force field. Proteins 78, 1950–1958 (2010). 20408171 10.1002/prot.22711 PMC2970904

[b75] Pérez A. . Refinement of the amber force field for nucleic acids: improving the description of *α/*γ conformers. Biophys. J. 92, 3817–3829 (2007). 17351000 10.1529/biophysj.106.097782 PMC1868997

[b76] Do T. N., Carloni P., Varani G. & Bussi G. *RNA/P*eptide binding driven by electrostatics? Insight from bidirectional pulling simulations. J. Chem. Theory Comput. 9, 1720–1730 (2013). 26587630 10.1021/ct3009914

[b77] Haider S. & Neidle S. Molecular Modeling and Simulation of G-Quadruplexes and Quadruplex-Ligand Complexes, 17–37 (Humana Press, Totowa, NJ, 2010). 10.1007/978-1-59745-363-9_2 20012413

[b78] Pigache A., Cieplak P. & Dupradeau F. Automatic and highly reproducible *RESP* and *ESP* charge derivation: Application to the development of programs red and x red. In Abstr. Pap. Am. Chem. Soc. vol. 227, U1011–U1011 (AMER Chemical SOC 1155 16TH ST, NW, Washington, DC 20036 USA, 2004).

[b79] Dupradeau F.-Y. . The red. tools: Advances in resp and esp charge derivation and force field library building. Phys. Chem. Chem. Phys. 12, 7821–7839 (2010). 20574571 10.1039/c0cp00111b PMC2918240

[b80] Cornell W. D. . A second generation force field for the simulation of proteins, nucleic acids, and organic molecules. J. Am. Chem. Soc. 117, 5179–5197 (1995).

[b81] Giberti F., Salvalaglio M., Mazzotti M. & Parrinello M. Insight into the nucleation of urea crystals from the melt. Chem. Eng. Sci. 121, 51–59 (2015).

[b82] Berendsen H. J., Postma J. v., van Gunsteren W. F., DiNola A. & Haak J. Molecular dynamics with coupling to an external bath. J. Chem. Phys. 81, 3684–3690 (1984).

[b83] Bussi G., Donadio D. & Parrinello M. Canonical sampling through velocity rescaling. J. Chem. Phys. 126, 014101 (2007). 17212484 10.1063/1.2408420

[b84] Hess B., Bekker H., Berendsen H. J. C. & Fraaije J. G. E. M. Lincs: A linear constraint solver for molecular simulations. Journal of Computational Chemistry 18, 1463–1472 (1997).

