# Supplementary material for: Focus on PNA Flexibility and RNA Binding using Molecular Dynamics and Metadynamics
Source: Sci Rep. 2017 Feb 17;7:42799. doi: 10.1038/srep42799 (PMC5314342; doi:10.1038/srep42799)
Supplement: Supplementary Information [file srep42799-s1.pdf]

SUPPLEMENTARY INFORMATION

Focus on PNA Flexibility and RNA Binding using Molecular Dynamics and Metadynamics

Massimiliano Donato Verona[1], Vincenzo Verdolino[2,3,*], Ferruccio Palazzesi[2,3], and Roberto Corradini[1,4,*]

[1]Dipartimento di Chimica, University of Parma, Italy, 43124,Italy

[2]Department of Chemistry and Applied Biosciences, ETH Zurich, c/o Universita` della Swizzera Italiana Campus, 6900 Lugano, Switzerland

[3]Facolta` di Informatica, Instituto di Scienze Computazionali, Universita` della Svizzera Italiana, 6900 Lugano, Switzerland

[4]National Institute for Biostructures and Biosystems (INBB)-Viale delle Medaglie d'Oro, 305, 00136 Roma, Italy

*Vincenzo Verdolino +41 058666 4809, email vincenzo.verdolino@phys.chem.ethz.ch

*Roberto Corradini +39 0521 905410, email roberto.corradini@unipr.it

CONTENTS

SI1 ssPNA flexibility pp. 2

SI1a RMSD trend of ssPNA during 200 ns long MD simulation

SI1b: FES local minima convergence

SI2 γ-ssPNA flexibility 4

SI2a RMSD trend of modified γ-ssPNA during 200 ns long MD simulation

SI2b: FES local minima convergence

SI2c: *ssPNA and γ-ssPNA structural analysis*

SI3 – Re-annealing simulations on ssPNA 8

SI3c H-bonding Fraction

SI4 – MD equilibrated PNA:RNA structure from data bank 10

SI5 – MD equilibrated γ-modified PNA:RNA structure 11

SI6 - Force field validations 11

SI7 - Stacking Variable 15

SI1 ssPNA flexibility

SI1a RMSD trend of ssPNA during 200 ns long MD simulation

In Fig. SI1a we report the Root Mean Square Deviation of ssPNA during 200 ns long MD simulation in order to investigate possible conformational transition with respect the initial helical one.

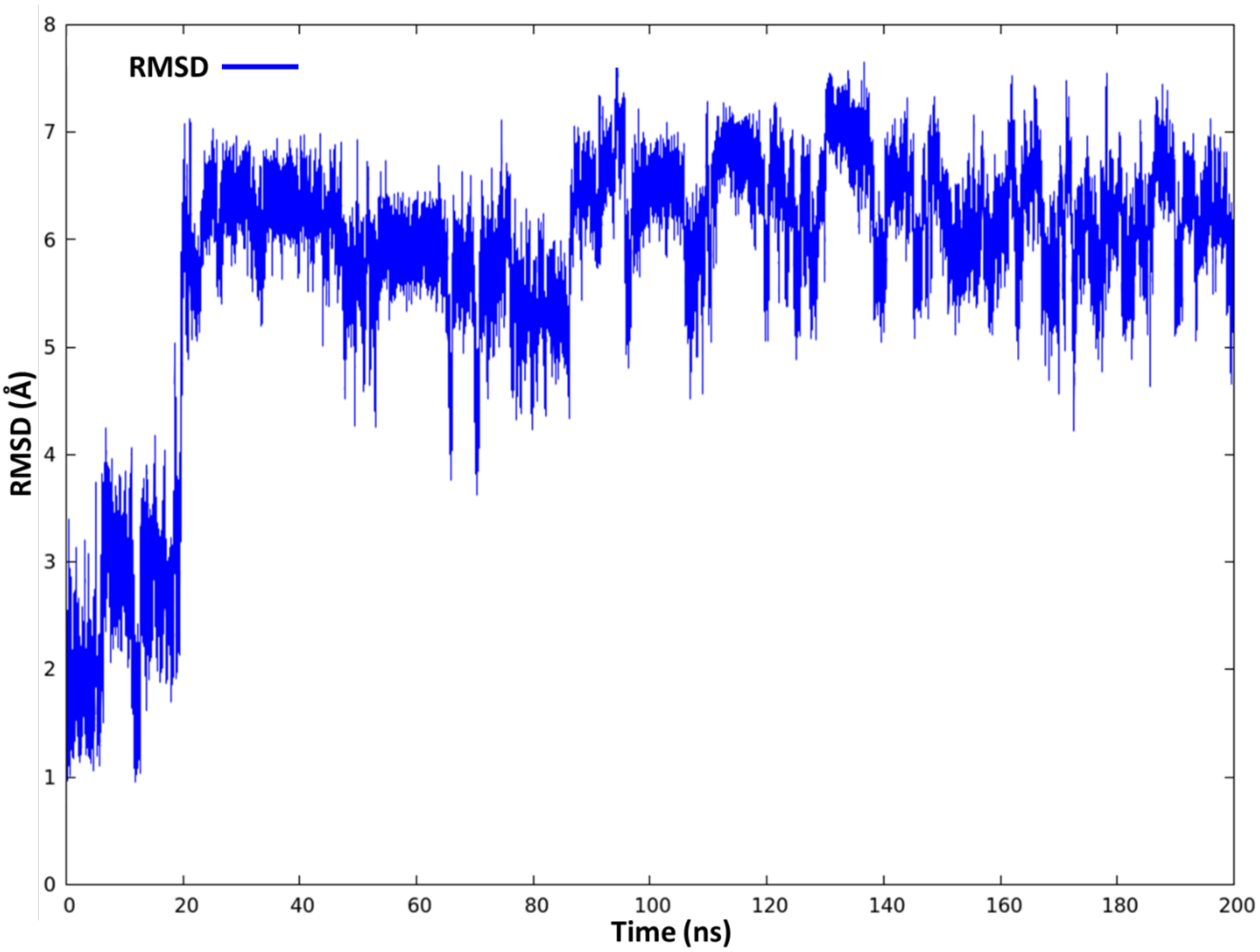

Fig. SI1a: RMSD plot along with the MD time for the unmodified ssPNA. The initial structure characterized by helical symmetry lasts for 20 ns and turns into a multitude of locally stable conformations till the end of the simulation.

SI1b: FES local minima convergence

In Fig. SI1b we report the local convergence analysis between the two minima A and B described in the main text. (Figure 2a) PDB Structures extracted from A and B available online

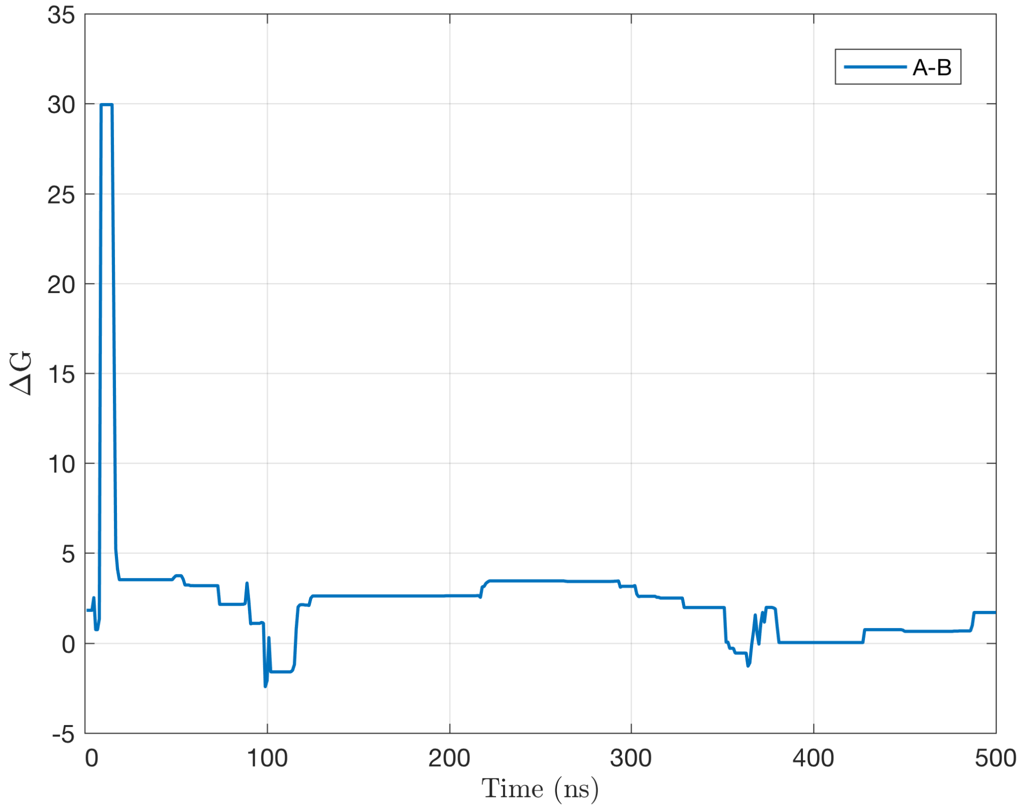

Fig. SI1b: Energy difference between A and B minima converged after 150 ns

SI2 γ-ssPNA flexibility

SI2a RMSD trend of modified γ-ssPNA during 200 ns long MD simulation

In Fig. SI2a we report the Root Mean Square Deviation of modified γ-ssPNA during 200 ns long MD simulation in order to investigate possible conformational transition with respect the initial helical one.

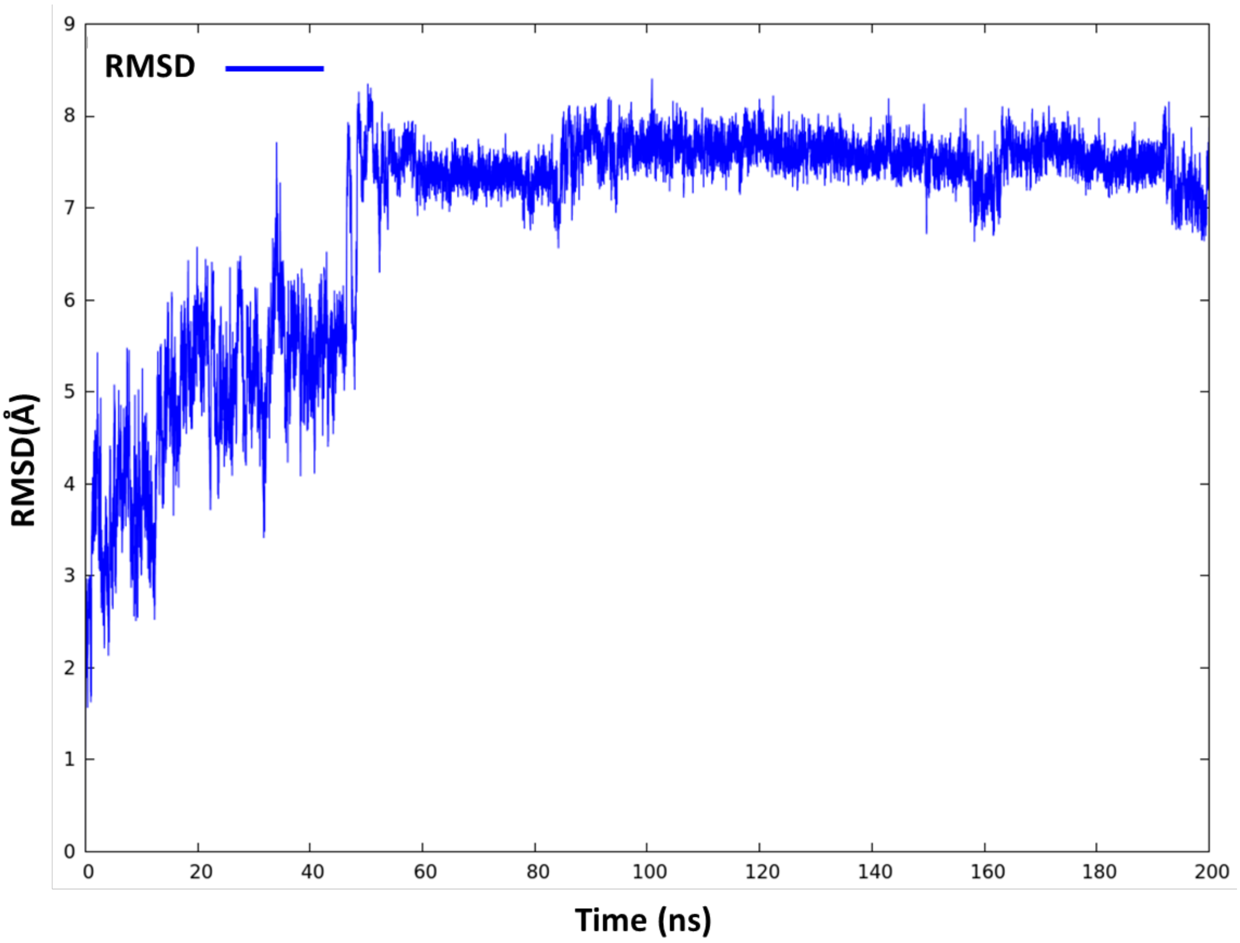

Fig. SI2a: RMSD plot along with the MD time for the modified γ-ssPNA. In this case the initial helical conformation is retained longer compared to ssPNA (55 ns instead of 20). The conformational displacement is smoother for the gamma modified and the RMSD fluctuation afterward considerably tighter denoting a higher degree of constraining and pre-organization.

SI2b: FES local minima convergence

In Fig. SI2b we report the local convergence analysis between four minima C-F as described in the main text. (Figure 2b) The convergence criteria is more flexible than the one employed in SI1b but, due to the large configurational space explored and the larger number of local minima characterizing the FES we can consider qualitatively acceptable these results. In particular we show the recrossing between states (top left), the head to tail (H-T) collective variable behaviour (top right), the sequential stacking recrossing (bottom left) and lastly the local free energy convergence between these four states along 400 ns. We recognize the difficulties in quantitatively converging the FES mainly due to the exploration of the stacking collective variable. However, these results show that WT-MDMT simulations extensively explored the entire conformational space visiting, in several different events, the principal structure clusters (C-F). PDB Structures extracted from C-F and representative videos (files beginning.mov "Supplementary Video S1: Beginning configuration of γ-ssPNA of WT-MDMT", and close.mov "Supplementary Video S2: Close configuration of γ-ssPNA during WT-MDMT" available online) of WT-MDMT trajectory available online.

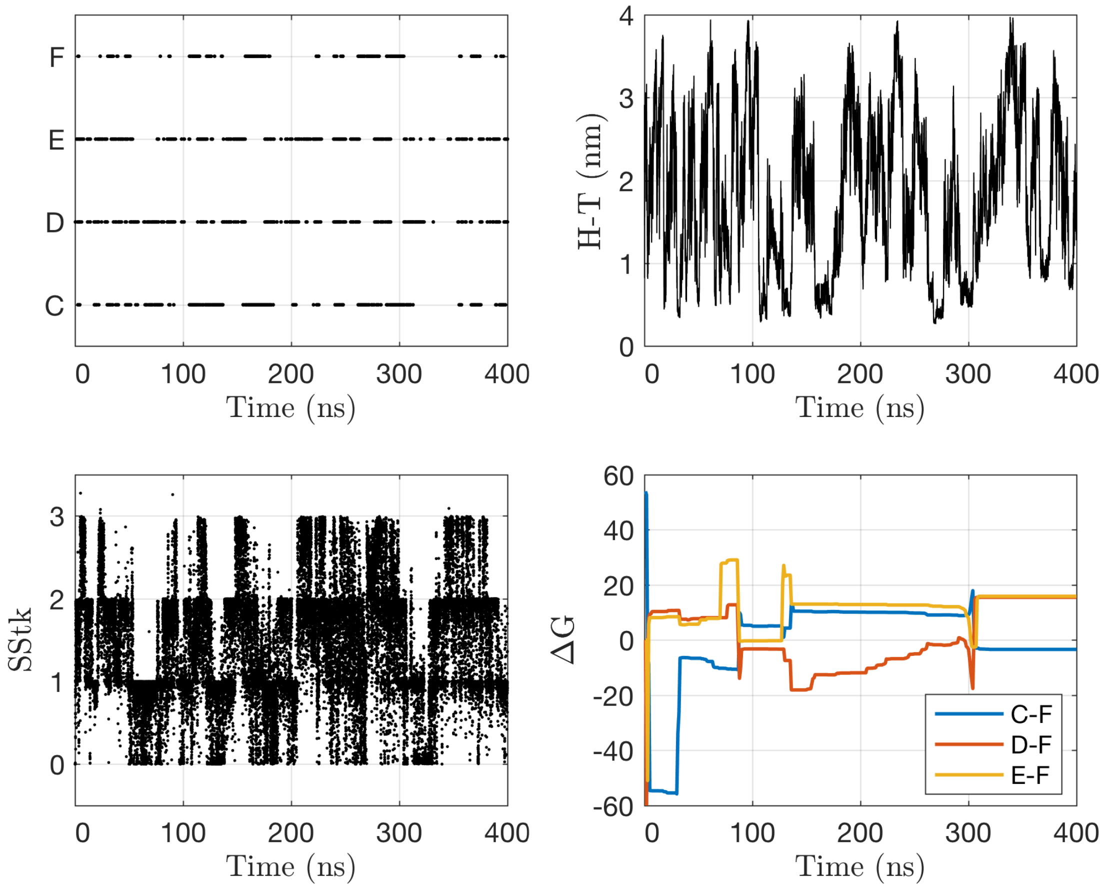

Fig. SI2b: Recrossing and energy differences between most important C-F local minima

Supplementary Video S1: Beginning configuration of γ-ssPNA of WT-MDMT

Supplementary Video S2: Close configuration of γ-ssPNA during WT-MDMT

SI2c: ssPNA and γ-ssPNA structural analysis

The torsion angles H-N-C-H_γ adjacent to the inter-residue amide bond on structures A-F reported in figure 2 (main text) have been monitored according to the following scheme SI2c, and comparing the corresponding torsion angle formed by the *pro*-R hydrogen in the corresponding monomer in the achiral ssPNA.

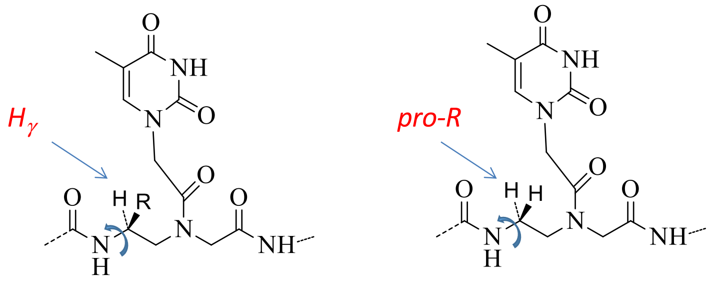

Scheme SI2c: Torsion angles considered for evaluation of conformation of structures A-F in Fig.2

In Fig. SI2c we report the histogram (weighted for the bias) of the distance between the sequential α-amino acidic hydrogens (red arrows in figure) calculated for the ssPNA (red curve) and γ-ssPNA (green curve). The two series of histograms are based on the WT-MDMT free energies reported in the main text. (Figure 2a and b respectively) As expected for a preorganized single strand the average distances (d1-d5) calculated for γ-ssPNA are significantly shorter than those in ssPNA. Most notably, the histogram resulting from the unmodified PNA shows an appreciable broadening at shorter and longer distances. On the contrary, the statistical distribution calculated for the γ-ssPNA is always very narrow. The unique exception is represented by the extremity d5 where the two systems behave similarly.

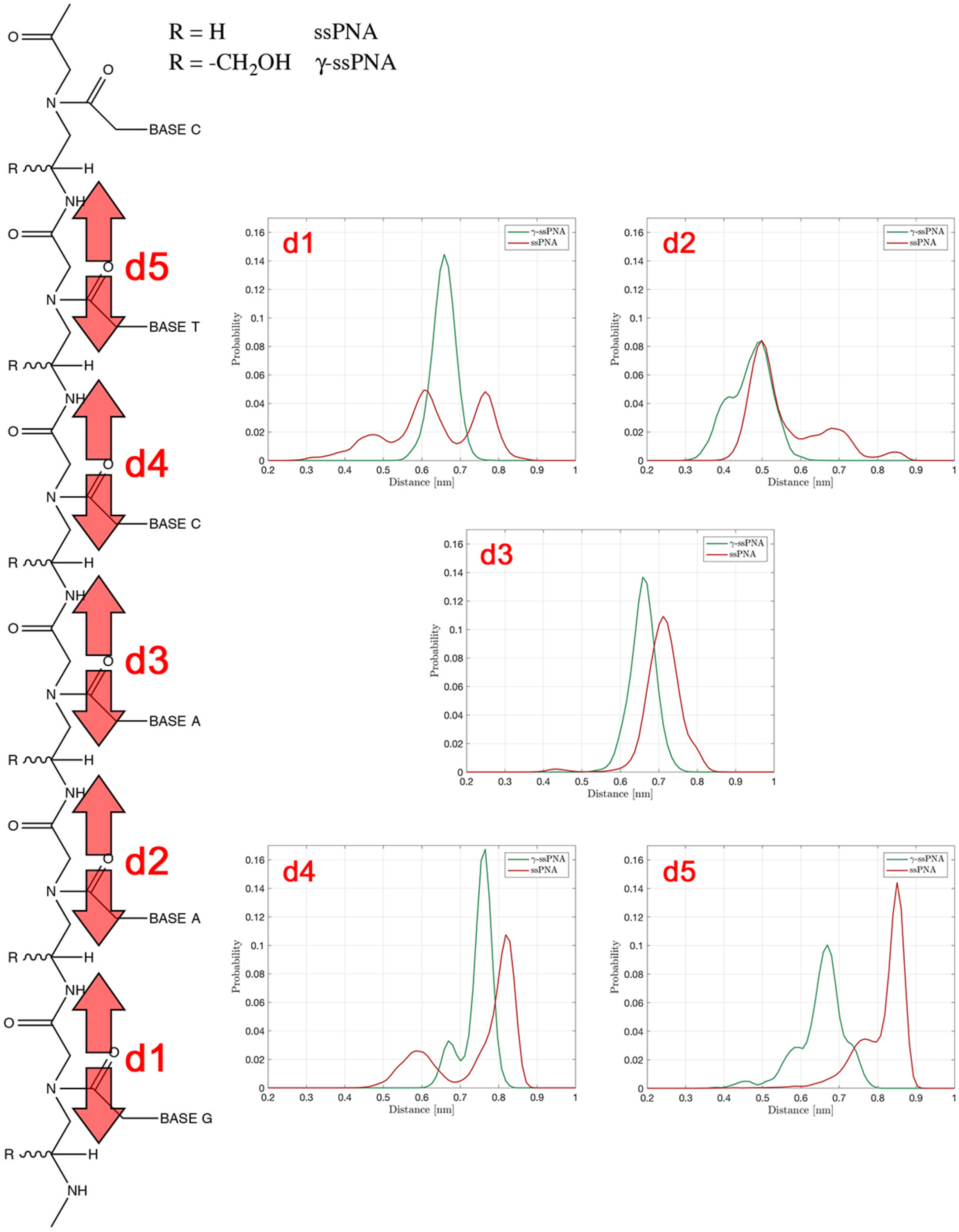

Fig. SI2c: Structural histogram analysis of the H--H distance of two sequential amino acids (d1-d5) for ssPNA (red) and γ-ssPNA (green)

SI3 – Re-annealing simulations on ssPNA

The PNA:RNA system considered in this study present the following sequences:

N'-GAACTC-C'

3'-CTTGAG-5'

In figure SI3a we report the base pairing recorded in 200 ns long MD simulations for all 5 different re-annealing.

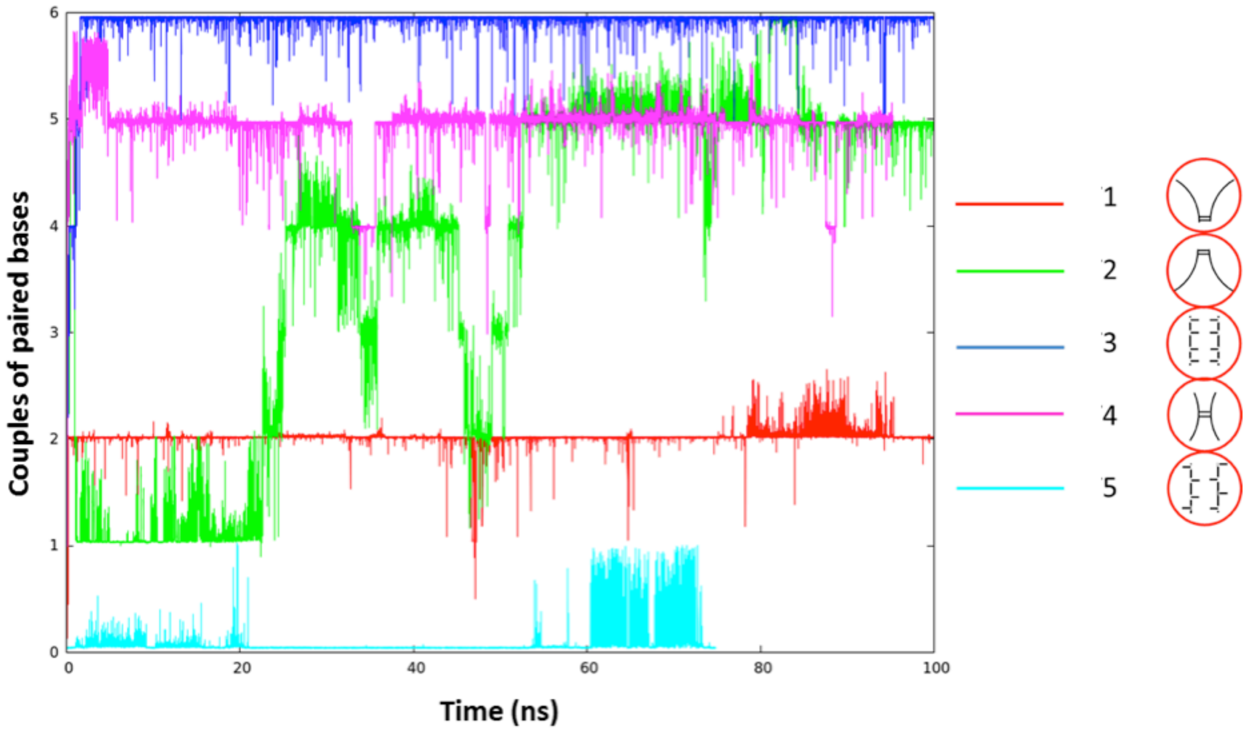

Fig. SI3a: Number of base paired as function of time for simulations **1-5** systems (structures reported in figure 4 of the main text).

In figure SI3b we present the base pairing as a function of time for simulations **2-4** (structures reported in figure 4 of the main text) focusing on selected bases as described in picture. Pairing in central bases resulted determinant for inducing higher level of duplex re-annealing.

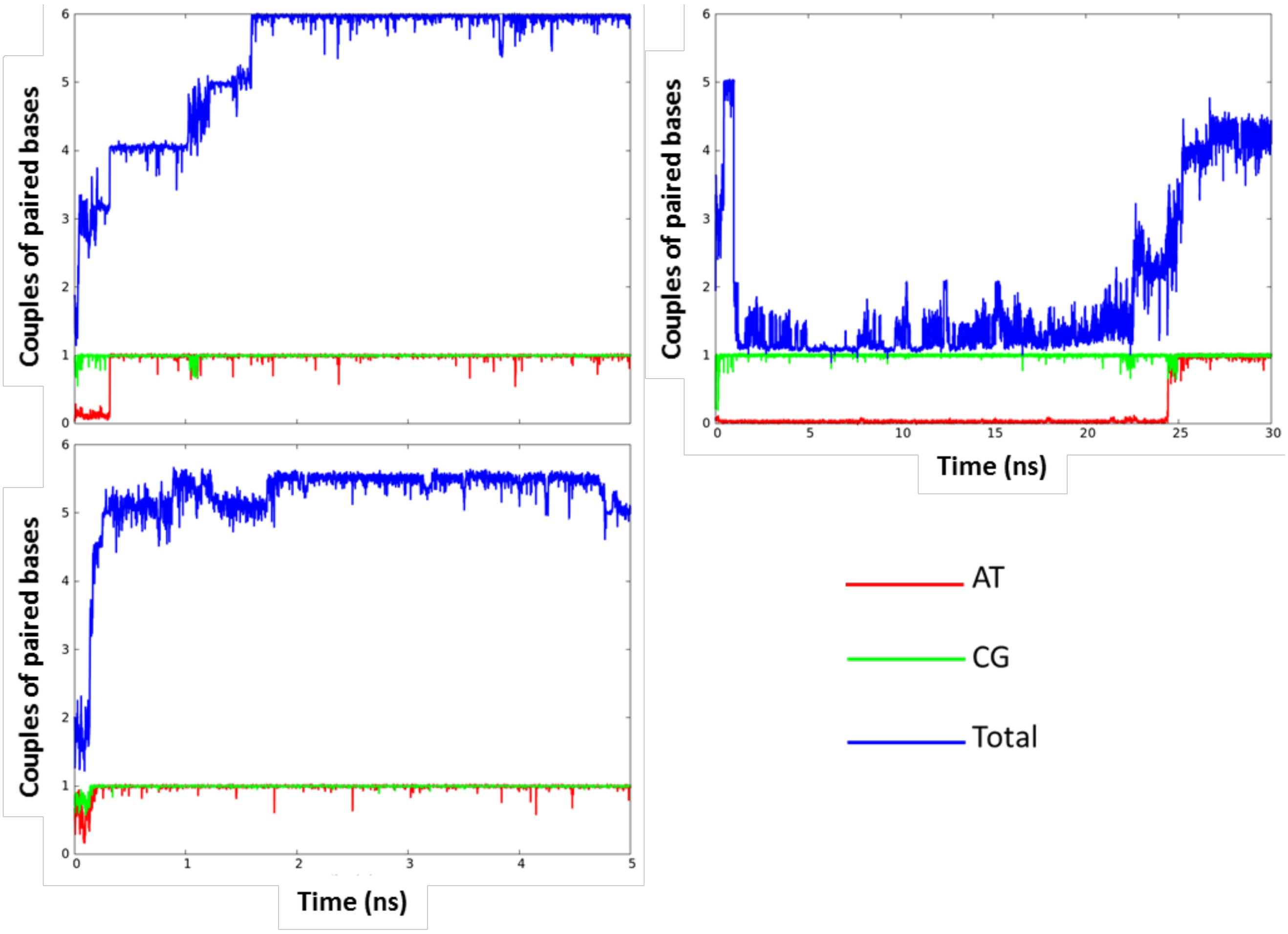

Fig. SI3b: Number of coupled bases as function of time for **2 (top left), 3 (top right) and (bottom left)** systems (structures reported in figure 4 of the main text). In red is represented **AT** couple base pairing, in green **CG** one and in blue the total pairing of the duplex.

SI3c H-bonding Fraction

The complete disruption of the helical structure is achieved when the inter-molecular base pairing becomes ineffective. In order to track this phenomenon with our simulations we calculate the h-bonding parameter using the “coordination function” collective-variable as implemented in *PLUMED*2. For each base, this quantity ranges from 0 to 1 depending on the effective h-bonding interaction. Considering the whole PNA:RNA structure, the total pairing ranges from 0 (completely disrupted) to 6 (optimal pairing). The ratio between the recorded and the theoretical h-bonding at a given time frame defines the h-bonding fraction. Of particular interest is the h-bonding fraction calculated just before the duplex disruption as reported in the main article. We tested a wide range of time frames for calculating the h-bonding fraction just before the duplex disruption. In a sufficiently narrow time range (~10 ns before disruption) the h-bonding fraction is independent on this choice. We selected for the results reported in the main text 0.5 ns as the best time frame for h-bonding fraction calculation.

SI4 – MD equilibrated PNA:RNA structure from data bank

In Fig. SI4 we represent the minimized and thermally equilibrated structure of the unmodified PNA:RNA described in the article. This structure is used as the starting point for the MD and WT-MDMT simulations.

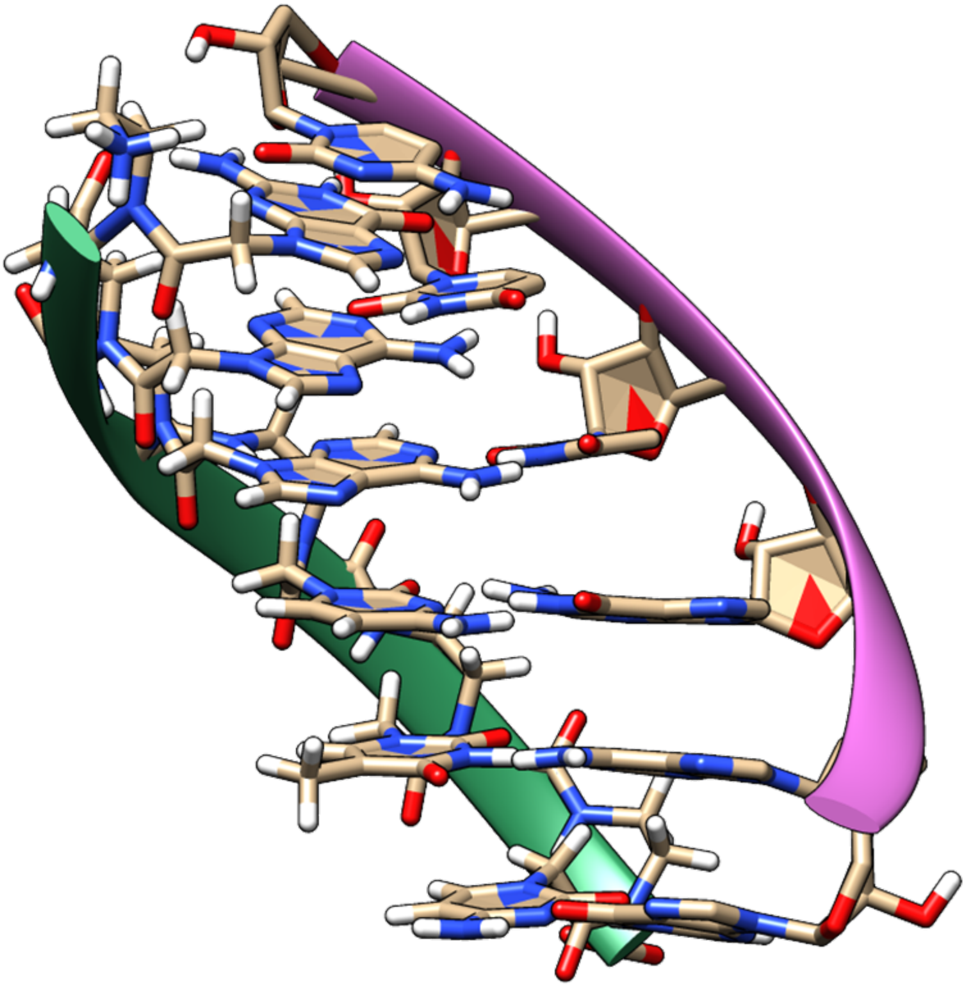

Fig. SI4: Thermally equilibrated structure of PNA:RNA duplex (176D), taken from Protein Data Bank and successively used for our simulations.

SI5 – MD equilibrated γ-modified PNA:RNA structure

In Fig. SI5 we represent the minimized and thermally equilibrated structure of the modified PNA:RNA described in the article. This structure is used as the starting point for the MD and WT-MDMT simulations.

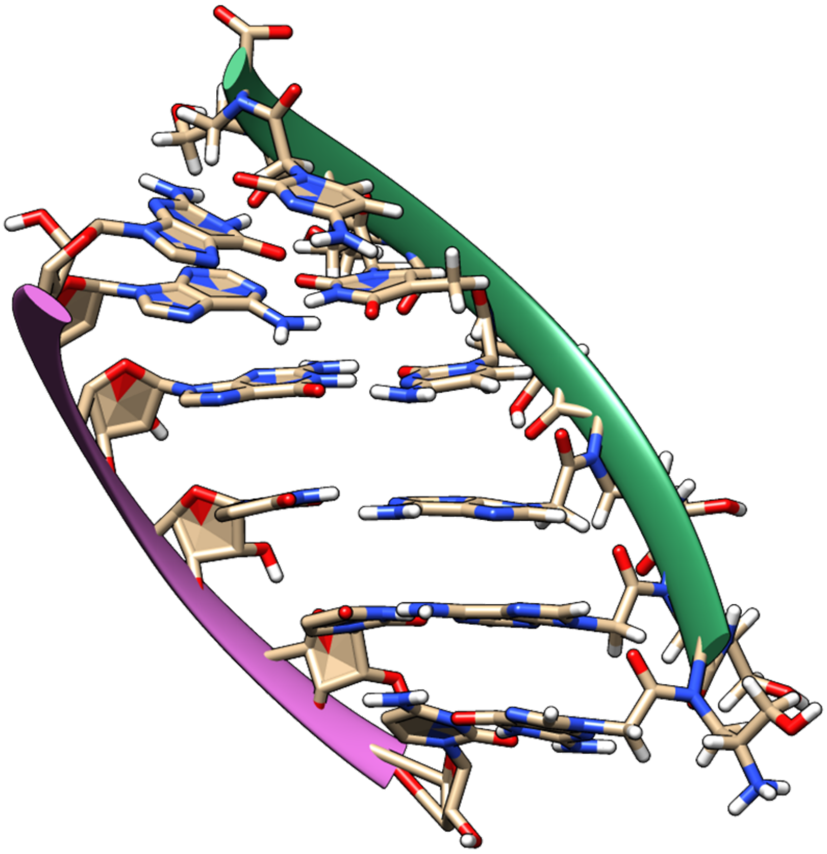

Fig. SI5: Structure of γ-modified PNA:RNA. The duplex was generated from system 176D retrieved from Protein Data Bank, by manual insertion of serine side chain in gamma (C5) position, and subsequently thermally equilibrated.

SI6 - Force field validations

As a starting model for assessment of the force field for PNA duplexes, we have chosen the 176D[1] NMR structure (reported in the Protein Data Bank database), which is one of the few PNA:RNA duplex structures reported in literature at the time of this study (crystal structure of PNA:RNA duplex was resolved for the first time at the end of December 2015).[2] The sequences of PNA and RNA are respectively H-GAACTC-O^- and 5'-GAGTTC-3'. This duplex was modified by removal of phosphate group bound to 5' residue of RNA, because the chosen force field (*ff99sb*) recognizes the RNA strand only without that group. In the literature there are no consolidated force fields for PNA, and thus one of our aims was to improve their availability similarly to other biological cases as proteins or nucleic acids that are nowadays widely accepted. In order to test the parameters chosen[3] we

performed a 200 ns long Molecular Dynamics simulation on the PNA:RNA duplex described above, using the protocol described in the Methods section. The duplex conformation was stable for all the simulation length with no significant structural modification. This can be inferred by examining the root mean square deviation (RMSD) that is less than 2.5 Å (Fig. SI6a).

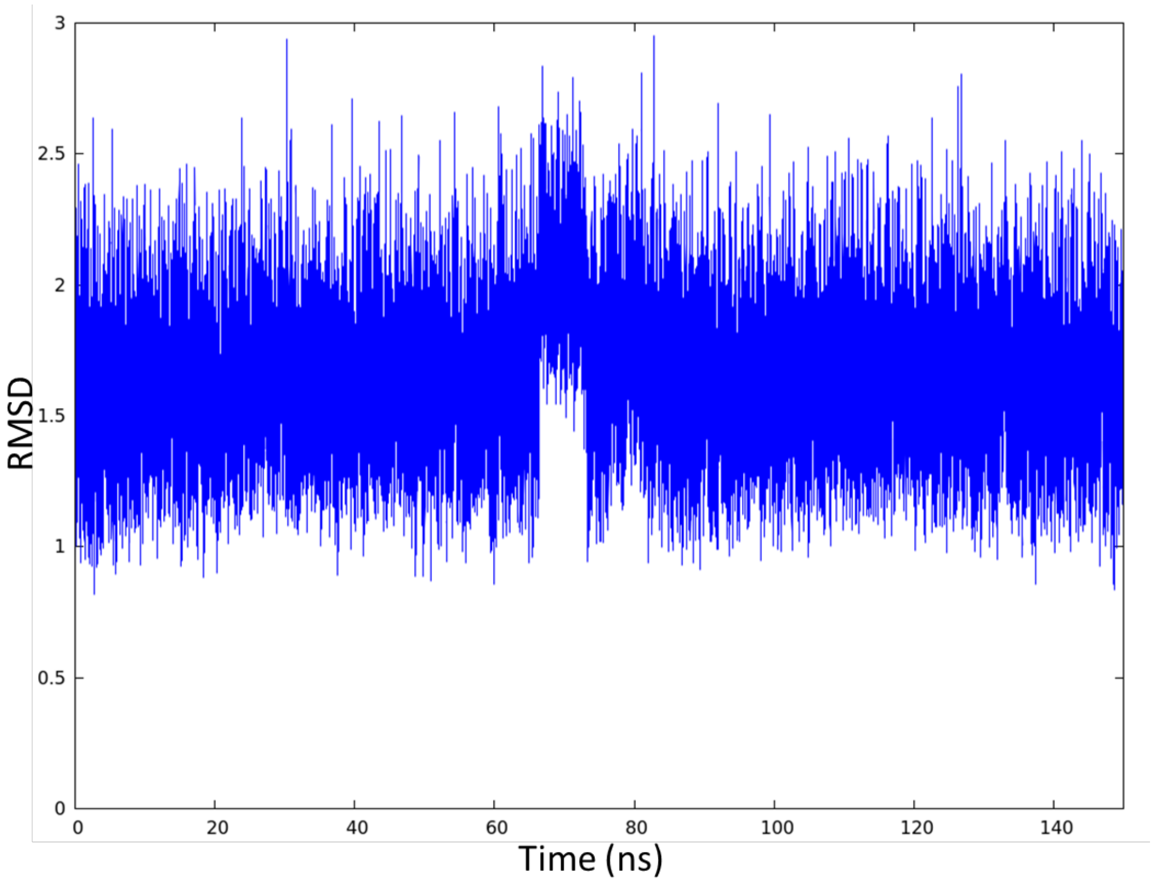

Fig. SI6a: RMSD plot of simulated PNA:RNA duplex as function of time.

To better test the force field we checked the characteristic torsion angles of PNA obtained in the MD simulation, compared with those reported in literature.[4] The results are in good agreement with the experimental ones (Fig. SI6b).

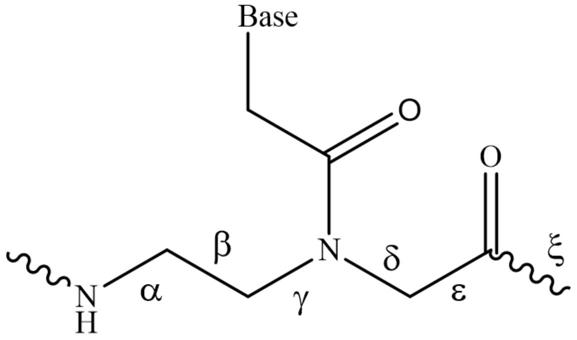

Angle	MD (°)	Experimental (°)
α	86.8±10.9	[40 - 100]
	250.8±15.1	[220 - 280]
β	70.7±11.2	[40 - 100]
γ	80.8±8.1	[60 - 100]
δ	95.6±9.8	[60 - 120]
ε	148.3±13.2	[130-180]
	339.2±16.9	[0 - 40;320 - 360]
ξ	170.3±12.1	[160 - 195]

Fig. SI6b: left, characteristic PNA angles; right, comparison between simulated angles and those reported in the literature.[4]

Next, we considered the MD simulation of γ-modified PNA with the force field developed. Also in this case we needed the force field was validated before using it for further investigations on PNA properties. Therefore, we performed a 50 ns long simulation on a duplex obtained by manual insertion of serine side chain in γ position of each monomer of the PNA:RNA duplex 176D used in the previous simulation. This calculation was done with a shorter simulation time because this system was expected to be even more stable than the unmodified one, according to the general properties of γ-modified PNA. Indeed, also in this simulation the duplex resulted perfectly paired for the entire simulation, as proven by the low RMSD (Fig. SI6c).

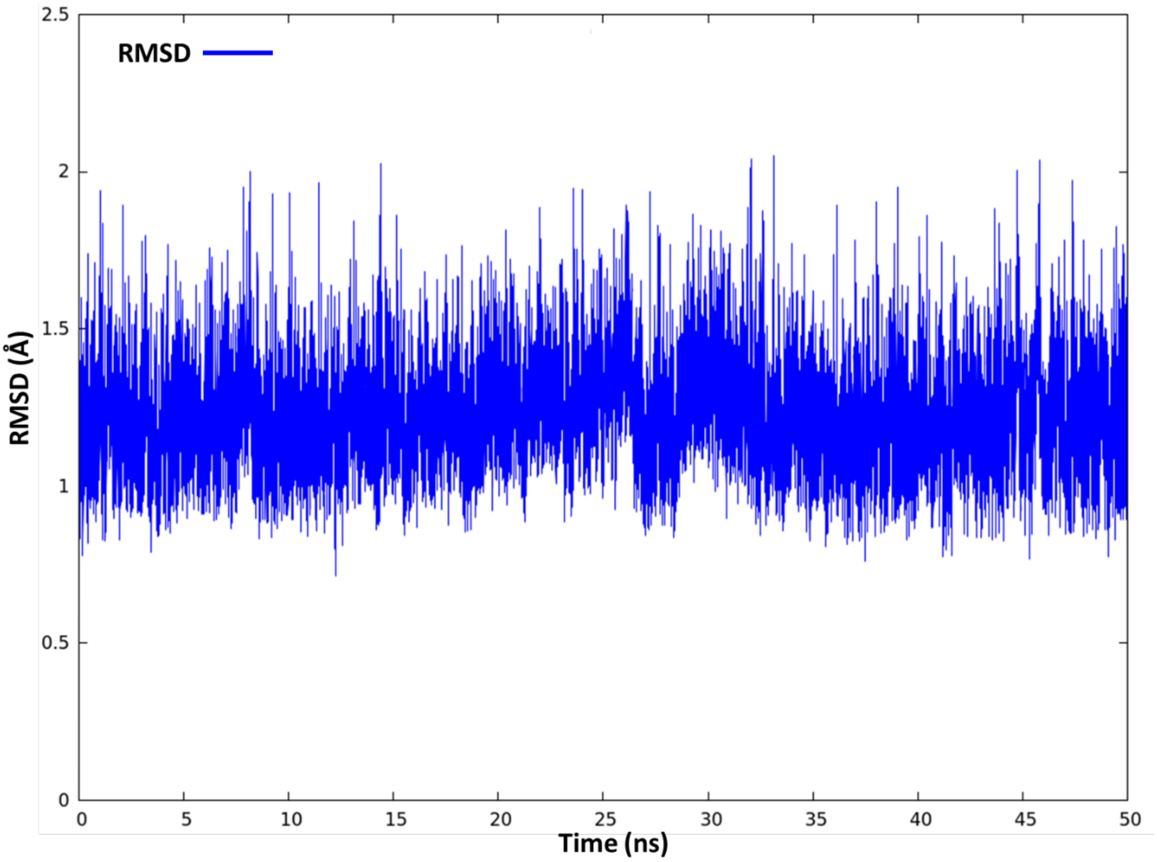

Fig. SI6c: RMSD plot of simulated γ PNA:RNA duplex as function of time.

Moreover, characteristic PNA angles were found also in this case to be compatible with those reported in literature (Fig. SI6d).

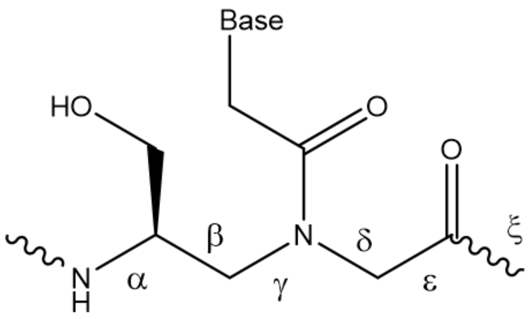

Angle	MD (°)	Experimental (°)
**α**	67.3±7.3	[40-100]
**β**	69.5±9.3	[40-100]
**γ**	81.1±7.9	[60-100]
**δ**	94.7±9.5	[60-120]
**ε**	165.8±10.3	[130-180]
**ξ**	170.1±8.1	[160-195]

Fig. SI6d: left, PNA bearing serine side chain in gamma position and its characteristic PNA angles; right, comparison between simulated angles and those reported in literature.[4,5]

SI7 - Stacking Variable

In order to better discriminate stacking stabilized conformations, we decided to use a local order parameter previously developed for describing crystal nucleation.[6] For each base, we defined a vector lying in the plane of the rings. This CV takes into account the distance, the angle and the coordination number between these vectors. Coordination number represents the number of vectors close within a defined cutoff. This parameter is important when studying nucleation since it determines the presence of an aggregate. The PNA considered in our study is 6 mer long and the maximum theoretical coordination number for each vector is then 5. However, our purpose is not to define an aggregate, but to discriminate stacking interactions. Therefore, for each couple of bases, we defined variables in order to have the coordination function $\boldsymbol{\rho_i}$ (equation **1.2**) always set to one, thus ruling out association. Distances and angles between these vectors are extremely important in stacking description: when two bases are stacked their distance should be defined in a given cutoff range and the angles should assume discrete values. An appropriate definition of this combination allows determining whether two or more bases are stacked (**SI7** A) or fully (**SI7** B – C) and partially unstacked (**SI7** D).

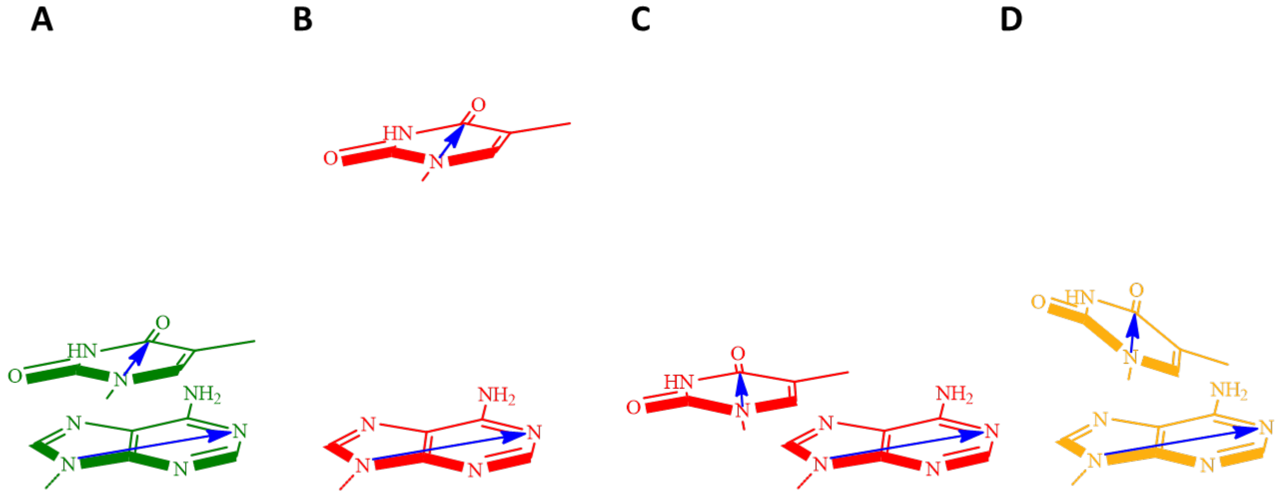

SI7: Schematic examples of stacking, not stacking and partial stacking arrangements. In blue are represented vectors used to describe local order parameter. **A**) Two bases are stacked; **B**) two bases are too distant for stacking interaction; **C**) two bases are close, but not stacked. In this case distance is favorable, but not the angle; **D**) Distance between bases is optimal, but angle not completely thus leading to partial stacking.

Going into the details, local order parameter is defined as the product of two sigmoidal and one single gaussian function:

$$\boldsymbol{f_{ij}} = \frac{\mathbf{1}}{\left(\mathbf{1}+e^{a\left(r_{ij}-r_{cut}\right)}\right)} \qquad \textit{\textbf{(1.1)}}$$

$$\boldsymbol{\rho_i} = \frac{\mathbf{1}}{\left(\mathbf{1}+e^{-b\left(n_i-n_{cut}\right)}\right)}$$

(Error! No text of specified style in document..1)

$$\theta_{ij} = \sum_{k=1}^{k_{max}} e^{-((\vartheta_{ij}-\vartheta_k)^2/2\sigma_k^2)} \quad \textbf{(1.2)}$$

where $\mathbf{r_{ij}}$ are the distances between the above discussed vectors, $\mathbf{r_{cut}}$ is cutoff distance for the stacking interaction, $\mathbf{n_i}$ is coordination number, $\mathbf{n_{cut}}$ is cutoff for coordination number, $\boldsymbol{\vartheta_{ij}}$ is the angle between the vectors, $\boldsymbol{\vartheta_k}$ is a favorable angle for stacking and $\boldsymbol{\sigma_k}$ is the width of the gaussian applied on that angle. Lastly, **a** and **b** are exponential factors, determining how steep are the sigmoids.

These functions essentially monitor respectively the distance between bases (equation **1.1**), the coordination number (equation **1.2**) and angle between bases (equation **1.3**). As discussed above, the coordination parameters were set to obtain a value of $\boldsymbol{\rho_i}$ = 1 ($\mathbf{n_{cut}}$ = 1). The distance function $\mathbf{f_{ij}}$ was set to have a value of 1 for distance within a range from 5.0 to 6.5 Å, depending on the couple of bases *i* and *j* considered. Above the cutoff distance, the $\mathbf{f_{ij}}$ value rapidly decreases to 0. The angle function $\boldsymbol{\theta_{ij}}$ is a sum of functions, one for each characteristic angle chosen. For each angle $\boldsymbol{\vartheta_k}$ a Gaussian function that exhibits maximum value of 1 for $\boldsymbol{\vartheta_k}$, is defined. In order to have stacking, bases should present opportune values of distances and angles. Based on MD simulation data, the coefficients ($\mathbf{r_{cut}}$, $\boldsymbol{\vartheta_k}$, $\boldsymbol{\sigma_k}$, **a**) were tuned in order to maximize $\mathbf{f_{ij}}$ and $\boldsymbol{\theta_{ij}}$ when bases are stacked. The functions here described are referred to a single couple of vectors, but in our system we have several possible couples and also multiple bases coupled at the same time. Description of every single couple is not meaningful alone, so to describe entirely the system we used a linear combination of these local order parameters, defined for couples of bases, and we considered this combination a measure of total stacking (**Stk**).

SI - Bibliography

1. Brown, S. C., Thomson, S. A., Veal, J. M. & Davis, D. G. NMR solution structure of a peptide nucleic acid complexed with RNA. *Science* **265,** 777–780 (1994).

2. Kiliszek, A., Banaszak, K., Dauter, Z. & Rypniewski, W. The first crystal structures of RNA-PNA duplexes and a PNA-PNA duplex containing mismatches-toward anti-sense therapy against TREDs. *Nucleic Acids Res.* 1513–1519 (2015). doi:10.1093/nar/gkv1513

3. REDDB Server. at <http://upjv.q4md-forcefieldtools.org/REDDB/projects/F-93/>

4. Topham, C. M. & Smith, J. C. The influence of helix morphology on co-operative polyamide backbone conformational flexibility in peptide nucleic acid complexes. *J. Mol. Biol.* **292,** 1017–1038 (1999).

5. He, W. *et al.* The structure of a gamma-modified peptide nucleic acid duplex. *Mol. Biosyst.* **6,** 1619–29 (2010).

6. Giberti, F., Salvalaglio, M., Mazzotti, M. & Parrinello, M. Insight into the nucleation of urea crystals from the melt. *Chem. Eng. Sci.* **121,** 51–59 (2015).
